# The assembly of mammalian SWI/SNF chromatin remodeling complexes is regulated by lysine-methylation dependent proteolysis

**DOI:** 10.1038/s41467-022-34348-9

**Published:** 2022-11-05

**Authors:** Pengfei Guo, Nam Hoang, Joseph Sanchez, Elaine H. Zhang, Keshari Rajawasam, Kristiana Trinidad, Hong Sun, Hui Zhang

**Affiliations:** 1 Department of Chemistry and Biochemistry, University of Nevada, Las Vegas, NV 89154 USA; 2 College of Natural Resources and College of Letters and Science, University of California, Berkeley, CA 94720 USA

**Keywords:** Ubiquitin ligases, Proteolysis

## Abstract

The assembly of mammalian SWI/SNF chromatin remodeling complexes is developmentally programed, and loss/mutations of SWI/SNF subunits alter the levels of other components through proteolysis, causing cancers. Here, we show that mouse *Lsd1/Kdm1a* deletion causes dramatic dissolution of SWI/SNF complexes and that LSD1 demethylates the methylated lysine residues in SMARCC1 and SMARCC2 to preserve the structural integrity of SWI/SNF complexes. The methylated SMARCC1/SMARCC2 are targeted for proteolysis by L3MBTL3 and the CRL4^DCAF5^ ubiquitin ligase complex. We identify SMARCC1 as the critical target of LSD1 and L3MBTL3 to maintain the pluripotency and self-renewal of embryonic stem cells. L3MBTL3 also regulates SMARCC1/SMARCC2 proteolysis induced by the loss of SWI/SNF subunits. Consistently, mouse *L3mbtl3* deletion causes striking accumulation of SWI/SNF components, associated with embryonic lethality. Our studies reveal that the assembly/disassembly of SWI/SNF complexes is dynamically controlled by a lysine-methylation dependent proteolytic mechanism to maintain the integrity of the SWI/SNF complexes.

## Abstract (web summary)

Here the authors show the assembly/disassembly of mammalian SWI/SNF complexes is dynamically regulated by a lysine methylation mechanism involving the demethylase LSD1 and the methyl-lysine reader L3MBTL3, which recognizes SET7-methylated lysines in SMARCC1 and SMARCC2 to target them for CRL4 ubiquitin ligase-mediated proteolysis.

## Introduction

The mammalian SWI/SNF (mSWI/SNF, also called BRG1/BRM-Associated Factor, BAF) complexes are large ATP-dependent chromatin remodeling complexes assembled from up to 15 subunits with multiple paralogues during development to regulate stem cells, differentiation, and cell fate determination^1,2^. The developmentally programed mSWI/SNF complexes assemble into cell/tissue-specific remodeling complexes by changing subunits or incorporating various subunit paralogues for the development of different tissues^3^. In pluripotent mouse embryonic stem cells (mESCs) and mouse F9 embryonic carcinoma cells, a unique mSWI/SNF complex, the esBAF complex, is assembled with limited key subunits, including BRG1 (SMARCA4 or BAF190A), the ATP-dependent chromatin remodeling catalytic subunit, SMARCC1 (BAF155 or SRG3), ARID1A (SMARCF1 or BAF250A), PBRM1 (Polybromo-1 or BAF180), and SMARCB1 (BAF47, INI1, or SNF5), to functionally associate with OCT4 and NANOG to regulate the central transcription circuitry of mESCs^4–7^. Loss of either BRG1 or SMARCC1 impairs the pluripotency and self-renewal of mESCs^4,5,7,8^, whereas ectopic expression of BRG1 or SMARCC1 in mouse embryonic fibroblasts (MEFs) significantly enhances SOX2, OCT4, and Myc-mediated reprogramming into induced pluripotent stem cells (iPSCs)^8^. When mESCs undergo differentiation, additional mSWI/SNF proteins, such as BRM (SMARCA2 or BAF190B), a BRG1 paralogue, and SMARCC2 (BAF170), a SMARCC1 paralogue, are co-expressed to form BRG1- and BRM-based SWI/SNF complexes^6^. In the mouse, homozygous deletions of *Brg1* or *Smarcc1* are early peri-implantation lethal during embryogenesis^9,10^, whereas *Brm* is dispensable and *Smarcc2* null mutant animals die shortly after birth^11^. Recent human cancer exome and whole-genome sequencing studies revealed that loss or mutations of mSWI/SNF genes encoding various subunits are associated with more than 20% of human cancers^2,3^. The mutations or loss of a single mSWI/SNF subunit gene often uniquely cause the proteolysis of other subunits to assemble aberrant mSWI/SNF complexes with changes in stoichiometry and composition^12–15^. Notably, cancer cells defective in a particular mSWI/SNF subunit are often vulnerable to synthetic lethality of losing its remaining paralogue in the residual SWI/SNF complexes^13–17^. For example, cancer cells defective in BRG1 are usually sensitive to the removal of BRM, whereas cancer cells with ARID1A mutations are vulnerable to the loss of ARID1B^13–17^. However, little is known about how the assembly or disassembly of mSWI/SNF complexes and the proteolysis of subunits are regulated during development and tumorigenesis.

Lysine methylation is an important post-translational protein modification and lysine residues in proteins can be mono-, di-, and trimethylated. Extensive research has established the key roles of various methylated lysine residues at the amino-terminal regions of histones in modulating chromatin structure and gene expression^18,19^. While trimethylations at lysines 9 and 27 in histone H3 (H3K9me3 and H3K27me3) are usually associated with repressive chromatin structure that suppresses gene expression, the trimethylation at lysine 4 in histone H3 (H3K4me3) typically associates with active transcription^18^. LSD1 (also called KDM1a) was originally identified as a histone demethylase that specifically removes methyl groups from the mono- and dimethylated lysine 4 in histone H3 (H3K4me1/me2), but not trimethylated H3K4, to repress transcription^18,20^. Null deletion of the mouse *Lsd1* gene causes early embryonic lethality around embryonic day 5.5 (E5.5)^21,22^. Loss of LSD1 also profoundly impairs the pluripotency and self-renewal of ESCs and the differentiation of other stem/progenitor cells, such as mouse hematopoietic stem cells^23–26^. The tamoxifen-induced conditional null deletion of the floxed *Lsd1* mice (4-9 weeks old) by actin-Cre-ER (CAGGCre-ER) in the hippocampal and cerebral cortex neurons leads to paralysis with widespread hippocampus and cortex neurodegeneration, as well as learning and memory defects^27^. However, the molecular targets of LSD1 deficiency that cause the devastating animal phenotypes remain unclear.

Many non-histone proteins, such as p53, DNA (cytosine-5)-methyltransferase 1 (DNMT1), SOX2, LIN28A, HIF1α, NFκB/RelA, ERα, GLI3, and E2F1, are monomethylated on specific lysine residues by SET7 (SET7/9, SET9, SETD7, or KMT7)^25,26,28–32^, which was originally isolated as a histone methyltransferase that mono-methylates H3K4^33,34^. Accumulating evidence indicates that methylation of lysine residues by SET7 on a group of non-histone proteins, such as DNMT1, E2F1, SOX2, NFκB/RelA, FOXO3, and STAT3^35–40^, triggers the proteolytic destruction of these modified proteins. We and others have recently shown that LSD1 acts as a demethylase to remove the methyl groups of monomethylated lysine residues in DNMT1, E2F1, and SOX2 proteins to prevent these proteins from the methylation-dependent proteolysis^25,26,37,41^. Here, we show that LSD1 also acts as a demethylase to regulate the assembly of mSWI/SNF complexes by preventing the proteolysis of mSWI/SNF subunits through a lysine methylation-dependent proteolytic mechanism.

## Results

**Fig. 1:**
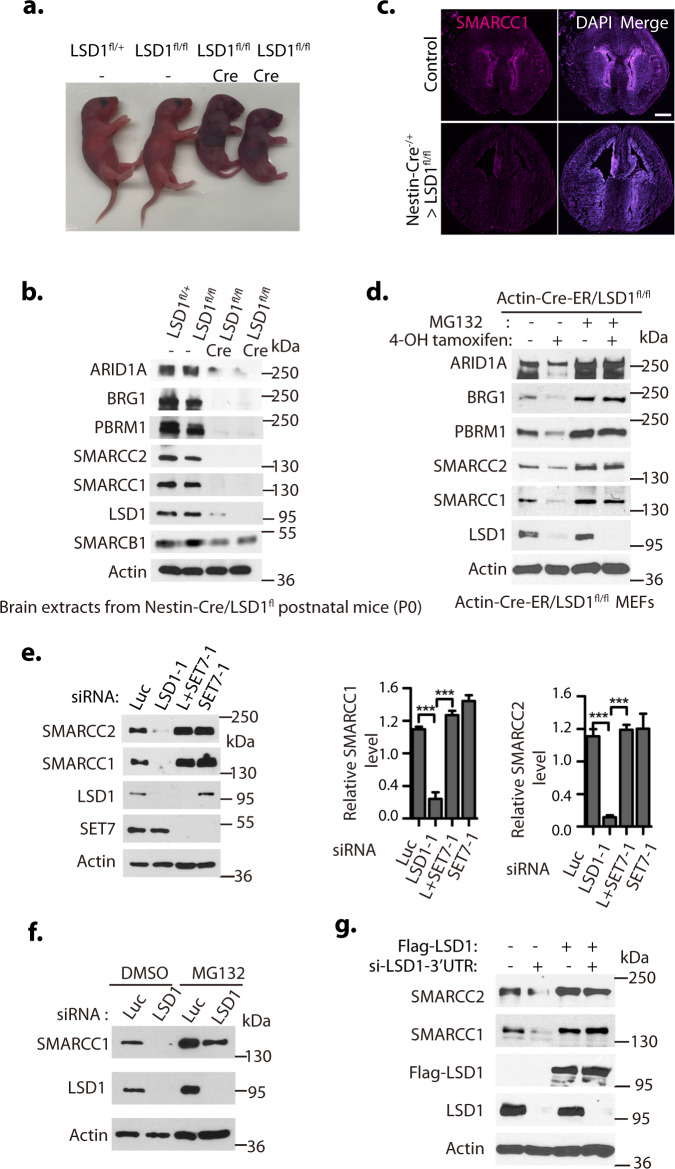
Dramatic loss of mSWI/SNF proteins in LSD1 null mice. **a** Nestin-Cre directed conditional inactivation of mouse *Lsd1* gene causes immediate postnatal death after birth (P0). The genomic DNA from the dissected brains from LSD1^flox/+^, LSD1^flox/flox^, and LSD1^flox/flox^/Nestin-Cre mutant mice immediately after birth were extracted and used for genome-typing. **b** Brain extracts from LSD1^flox/+^, LSD1^flox/flox^, and LSD1^flox/flox^/Nestin-Cre mice were Western blotted for indicated SWI/SNF proteins. Protein molecular weight markers are in kilodaltons (kDa). **c** Representative brain cryo-sections of postnatal LSD1^flox/flox^ and LSD1^flox/flox^/Nestin-Cre mice (P0,10-µm thick coronal) were stained with anti-SMARCC1 antibody, counter-stained with 4′,6-diamidino-2-phenylindole (DAPI). Scale bar: 500 μm. **d** Embryonic fibroblasts from CAGGCre-ER^TM^/LSD1^fl/fl^ mouse embryos (E13.5) were treated with 4-hydroxytamoxifen (20 μg/ml) for 12 h to delete *Lsd1* by inducible Actin-Cre-ER. Dimethyl sulfoxide (DMSO) or proteasome inhibitor MG132 (5 μg/ml) was added for the last 6 h before lysing the cells. **e** HeLa cells were transfected with 50 nM siRNAs of luciferase (Luc), LSD1, LSD1 + SET7 (L + SET7), or SET7 for 48 h and representative Western blots from three independent experiments are shown with Actin as a loading control. Band intensities were quantified by ImageJ software and normalized to the Luciferase (Luc) siRNA control signal. Significance was indicated as a two-tailed, unpaired, *t*-test. Values are expressed as the mean ± SEM. ***p < 0.001. **f** H1299 cells stably expressing Flag-tagged SMARCC1 under the retroviral LTR promoter control were transfected with 50 nM of luciferase and LSD1 siRNAs for 48 h and then treated with either dimethyl sulfoxide (DMSO, 1%) or 5 μg/ml MG132 for last 6 h before lysing the cells for Western blotting. **g** H1299 cells with or without stably expressing Flag-tagged LSD1 cDNA under the retroviral LTR promoter control were transfected with 50 nM of luciferase and LSD1-3’UTR siRNAs for 48 h and a representative blot set is shown for Western blotting. Source data are provided as a Source Data file. Significance was indicated as a two-tailed, unpaired, *t*-test. Values are expressed as the mean ± SEM. ***p < 0.001. The experiments for **a**–**d** and **f** were repeated three times with the same results.

To understand the molecular mechanism by which *Lsd1* null deletion causes embryonic lethality and other devastating effects in animals, we bred the floxed *Lsd1* conditional deletion mouse strain^24^ with the nestin-Cre transgenic mice^42^ to specifically delete *Lsd1* in the neuronal and glial cell precursors of the central nervous system. We found that the nestin-Cre-mediated homozygous loss of *Lsd1* conditional alleles caused postnatal lethality immediately after birth (P0, Fig. 1a). During the characterization of *Lsd1* mutants, we repeatedly observed that the protein levels of several subunits of mSWI/SNF chromatin remodeling complexes, such as BRG1, SMARCC1, SMARCC2, ARID1A, and PBRM1, are dramatically reduced in the brain extracts of *Lsd1* null mutants, but not in the extracts from the body parts that do not express nestin-Cre, as compared with that of wildtype littermates (Fig. 1b and Supplementary Fig. 1b). The dramatic reduction of these mSWI/SNF subunit protein levels occurs post-transcriptionally, as there is no significant correlative differences at the mRNA levels of these mSWI/SNF subunits between the *Lsd1* null mutants and the wildtype littermates in the brain tissues (Supplementary Fig. 1a). Consistently, our immunostaining of brain sections from the wildtype and *Lsd1* null mutant animals revealed that the protein level of SMARCC1, a core mSWI/SNF component^14,43–45^, is markedly reduced in the *Lsd1* mutant mice (Fig. 1c). To rule out potential degradation of mSWI/SNF proteins caused by animal lethality, we bred *Lsd1* conditional deletion mouse strain^24^ with a transgenic mouse line expressing a tamoxifen inducible Cre-ER recombinase under the Actin promoter control (CAGGCre-ER)^46^ to establish mouse embryonic fibroblasts (MEFs) from the homozygous floxed LSD1 conditional (fl/fl) deletion mouse embryos with the actin-Cre-ER (CAGGCre-ER/*Lsd1*fl/fl). While these MEFs normally express significant levels of mSWI/SNF proteins, induced deletion of *Lsd1* in the CAGGCre-ER/*Lsd1*fl/fl MEFs by addition of 4-hydroxytamoxifen (4-OH-Tam) led to the rapid disappearance of these mSWI/SNF proteins (Fig. 1d). The 4-OH-Tam induced downregulation of mSWI/SNF proteins was reversed by the treatment of these *Lsd1* deficient MEFs with 26 S proteasome inhibitor, MG132 (Fig. 1d). To further confirm that the protein stability of mSWI/SNF subunits is dependent on *Lsd1*, we treated the CAGGCre-ER/*Lsd1*fl/fl MEFs with a specific LSD1 inhibitor, CBB3001 that we previously developed^41,47^. The protein levels of mSWI/SNF subunits decreased substantially within 10 h of CBB3001 treatment (Supplementary Fig. 1c). Our studies indicate that LSD1 is required to maintain the protein stability and integrity of mSWI/SNF complexes.

### SMARCC1 and SMARCC2 are targets of LSD1

Mutations or loss of a single critical mSWI/SNF subunit, such as SMARCB1 in malignant rhabdoid tumors and epithelioid sarcoma, ARID1A in a wide variety of cancers including ovarian clear cell carcinoma, endometrioid carcinoma, neuroblastoma, and bladder cancer, BRG1 in medulloblastoma, breast and lung cancers, SMARCC1 in small cell lung cancer, and SMARCC2 in pancreatic cancer, uniquely causes the proteolysis of other subunits^2,3,12–15^. Recent studies indicate that the structural integrity of the mSWI/SNF complexes requires the formation of homo- or heterodimer of SMARCC1 and SMARCC2^43^, and loss of SMARCC1 and/or SMARCC2 causes the disassembly of mSWI/SNF complexes and proteolytic degradation of many other mSWI/SNF subunits^15,44,45^. As LSD1 serves as a demethylase for DNMT1, E2F1, and SOX2^25,26,41^, we wondered whether mSWI/SNF proteins are regulated by lysine methylation. While lysine methylations of mSWI/SNF proteins have not been reported, our protein sequence examination revealed that SMARCC1 and SMARCC2 both contain several H3K4-like lysine residues that may be monomethylated by the SET7 methyltransferase (see below). To test whether SMARCC1 or SMARCC2 is regulated by SET7, we used siRNA-mediated silencing of SET7 with two representative siRNAs^41^ and found that loss of SET7 indeed increased the levels of both SMARCC1 and SMARCC2 proteins in human cervical carcinoma HeLa cells (Fig. 1e and Supplementary Fig. 1d). In addition, silencing of LSD1 with two representative siRNAs^41^ reduced the protein levels of SMARCC1 and SMARCC2, and co-silencing of SET7 and LSD1 effectively re-stabilized SMARCC1 and SMARCC2 proteins in LSD1 deficient cells (Fig. 1e and Supplementary Fig. 1d). To examine whether loss of LSD1 or SET7 affects the protein stability of SMARCC1 and SMARCC2, siRNA-transfected cells were treated with protein synthesis inhibitor cycloheximide to block translational initiation to measure protein decay rates^48^. We found that the half-lives of SMARCC1 and SMARCC2 proteins were reduced after LSD1 silencing. However, co-silencing of LSD1 and SET7 restored the half-lives of both SMARCC1 and SMARCC2 proteins analyzed by measuring the steady-state protein stability (Supplementary Fig. 2). We also stably expressed Flag-tagged SMARCC1 or SMARCC2 under the retroviral LTR promoter control^41^ in human lung carcinoma H1299 cells and found that the ectopically expressed Flag-SMARCC1 and SMARCC2 are also sensitive to LSD1 silencing (see below). The downregulation of SMARCC1 protein induced by LSD1 silencing is also reversed by 26 S proteasome inhibitor, MG132 (Fig. 1f)^25,26^. To exclude the potential off-target effect of siRNAs, we ectopically expressed Flag-tagged LSD1 cDNA that does not contain the 3′ untranslated region (UTR), and then silenced the expression of endogenous LSD1 using the siRNA targeting the 3′UTR region of the endogenous LSD1 gene in H1299 cells. We found that the ectopically expressed Flag-LSD1 cDNA suppressed the 3’UTR silencing effects of endogenous LSD1 on SMARCC1 and SMARCC2 proteins (Fig. 1g). These observations indicate that loss of LSD1 induces the proteolysis of SMARCC1 and SMARCC2 proteins through the ubiquitin-dependent pathway.

### L3MBTL3 regulates the proteolysis of SMARCC1 and SMARCC2

**Fig. 2:**
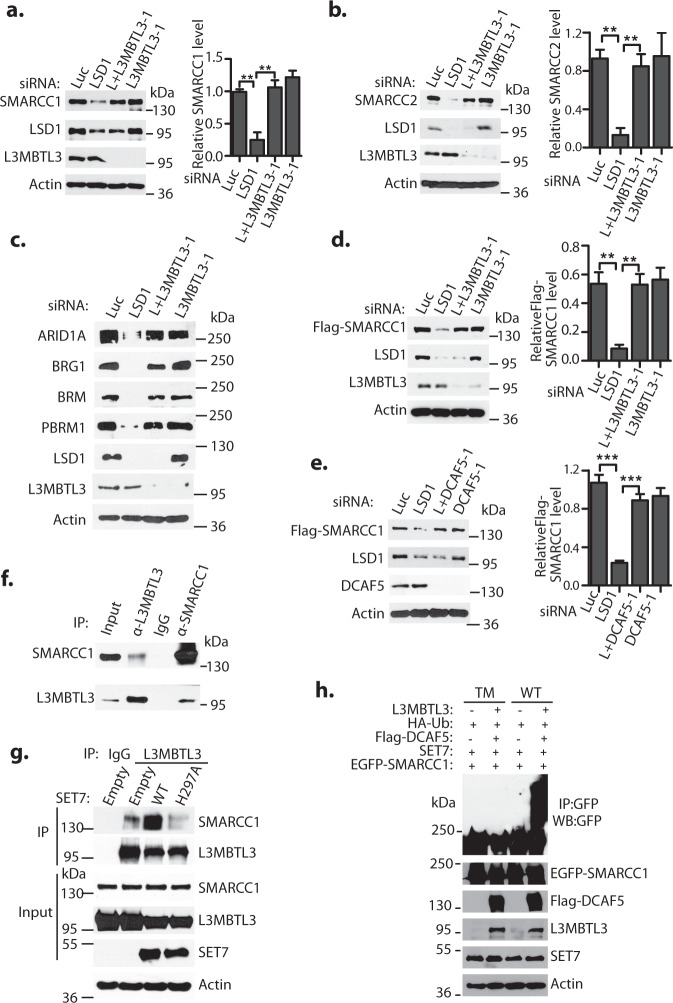
Regulation of SMARCC1 and SMARCC2 by L3MBTL3 and DCAF5. **a**–**c** L3MBTL3 silencing stabilizes endogenous mSWI/SNF proteins in HeLa cells. HeLa cells were transfected with 50 nM siRNAs of luciferase, LSD1, LSD1 + L3MBTL3 (L + L3MBTL3), or L3MBTL3 for 48 h. The representative levels of SMARCC1 (**a**), SMARCC2 (**b**), and other indicated mSWI/SNF proteins (**c**) were analyzed by Western blotting. Band intensities were quantified and normalized to the Luciferase (Luc) siRNA control signal, as described in Fig. 1e. **d** H1299 cells stably expressing Flag-SMARCC1 were transfected with 50 nM of luciferase, LSD1, LSD1 + L3MBTL3, and L3MBTL3 siRNAs for 48 h and Flag-SMARCC1 levels were analyzed by Western blotting. **e** DCAF5 silencing stabilizes Flag-SMARCC1. H1299 cells expressing Flag-SMARCC1 were transfected with 50 nM of luciferase, LSD1, LSD1 + DCAF5, and DCAF5 siRNAs and quantitatively analyzed as **2d**. **f** Endogenous L3MBTL3 interacts with SMARCC1. L3MBTL3 and SMARCC1 were immunoprecipitated from 293 T cells and blotted with respective antibodies. **g** SET7 stimulates L3MBTL3-SMARCC1 interaction. H1299 cells expressing Flag-SMARCC1 were transfected with control empty vector or the vectors expressing SET7 wildtype (WT) or the H297A mutant of SET7 for 48 h and cell lysates were immunoprecipitated by IgG control or anti-L3MBTL3 antibody. Western blotting was performed with antibodies against indicated proteins. **h** EGFP-tagged wildtype and triple mutant of SMARCC1 expressing constructs were co-transfected into 293 T cells together with vectors expressing HA-tagged ubiquitin (HA-Ub), and SET7 in the presence or absence of L3MBTL3 and DCAF5 expressing constructs as indicated. Proteins were immunoprecipitated with anti-GFP antibodies and Western blotted with the anti-GFP-SMARCC1 and other antibodies against indicated proteins. Source data are provided as a Source Data file. Significance was indicated as a two-tailed, unpaired, *t*-test for (**a**, **b**, **d**, **e**). Values are expressed as the mean ± SEM. **p < 0.01,***p < 0.001.

Since LSD1 is a demethylase, it is possible that SMARCC1 and SMARCC2 might be lysine methylated to trigger their proteolysis. We have recently shown that the monomethylated lysine residues in DNMT1, E2F1, and SOX2 are recognized by L3MBTL3, a Malignant Brain Tumor (MBT) domain protein that specifically binds to methyl-lysine residues^49^, to target substrate proteolysis by the CRL4^DCAF5^ ubiquitin E3 ligase complex^26,41^. We wondered whether L3MBTL3 and DCAF5, a substrate-specific subunit of CRL4 that interacts with L3MBTL3^41^, regulate the proteolysis of SMARCC1 or SMARCC2 in LSD1 silenced cells. Indeed, siRNA-mediated silencing of L3MBTL3 with two representative siRNAs^41^ stabilized both SMARCC1 and SMARCC2 proteins in LSD1 deficient cells (Figs. 2a, b and Supplementary Fig. 3a). Furthermore, while LSD1 silencing led to the downregulation of other mSWI/SNF proteins such as BRG1, BRM, ARID1A, and PBRM1, co-silencing of L3MBTL3 and LSD1 reversed these downregulations in LSD1 silenced cells (Fig. 2c and Supplementary Fig. 3c). Moreover, our immuno-co-precipitation analysis revealed that endogenous L3MBTL3 and SMARCC1 proteins reciprocally interact with each other (Fig. 2f), and this interaction is significantly enhanced in the presence of wild-type SET7, but not when a methyltransferase-deficient mutant of SET7, in which the critical histidine 297 is converted to alanine (H297A)^37^, is expressed (Fig. 2g). Conversely, downregulation of SET7 greatly reduced the interaction between L3MBTL3 and SMARCC1 (Supplementary Fig. 4a). In addition, while LSD1 silencing induced the degradation of ectopically expressed Flag-SMARCC1 protein, such degradation was prevented by co-silencing of L3MBTL3 or DCAF5 and LSD1 (Figs. 2d, e and Supplementary Fig. 3a, b). Either the expression of SET7 or the treatment of LSD1 silencing can also stimulate SMARCC1 polyubiquitination, which is further promoted by the co-expression of SET7, L3MBTL3, and DCAF5 (Fig. 2h and Supplementary Fig. 4b).

**Fig. 3:**
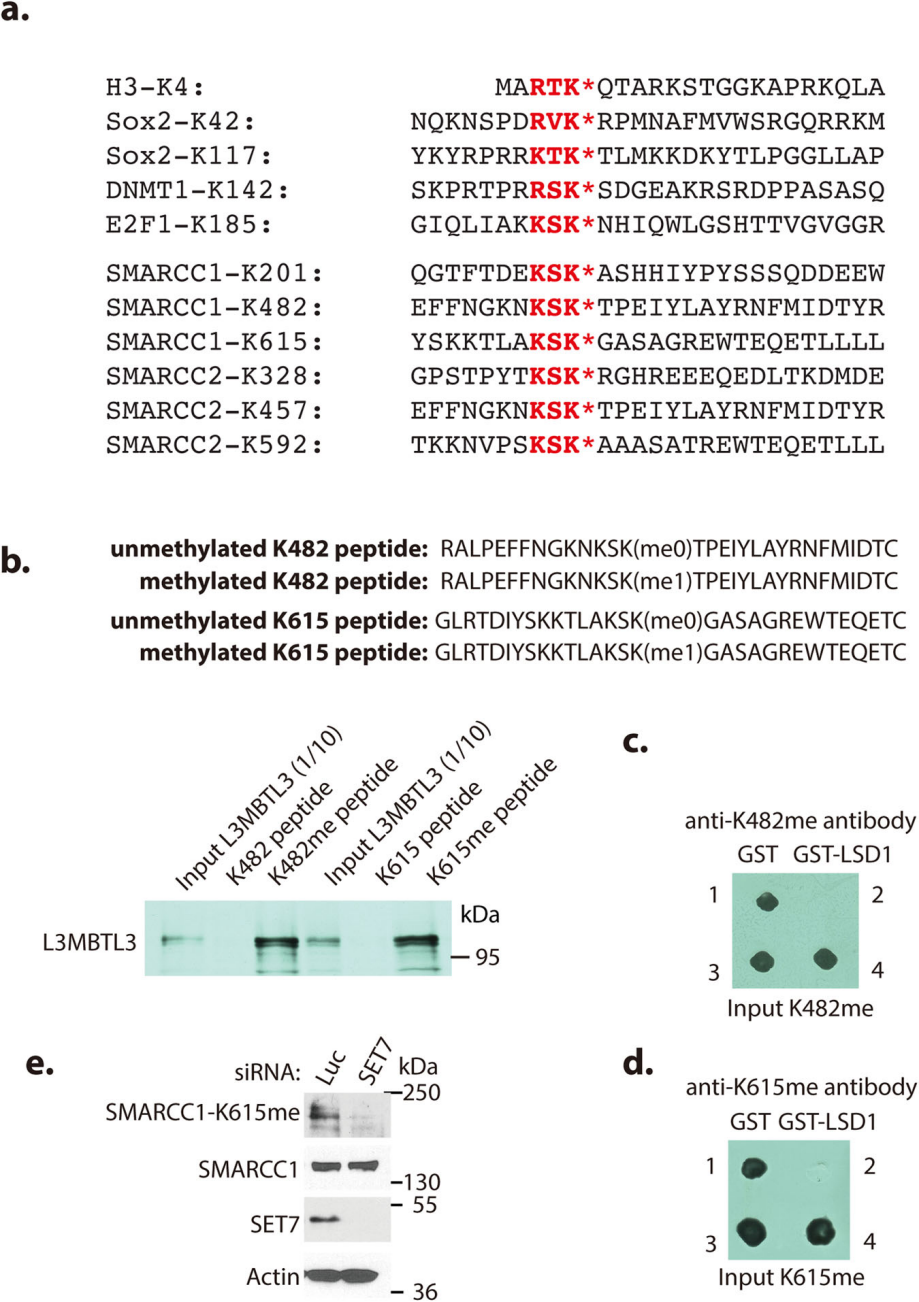
L3MBTL3 and LSD1 recognize the methylated K482 and K615 in SMARCC1. **a** The consensus lysine residues (K*) methylated by SET7 in H3K4-like methylation motifs with the R/K-S/T/V-K* consensus sequences. **b** L3MBTL3 preferentially binds to the monomethylated K482 and K615 peptides. Sulfolink-agarose immobilized SMARCC1 peptides containing the monomethylated K482 and K615, or cognate unmethylated peptides were incubated with 20 μl of in vitro translated and ^35^S-methionine labeled L3MBTL3 for 1 h at room temperature. L3MBTL3 binding to the peptide resins was analyzed by autoradiography after protein electrophoresis separation. **c**, **d** LSD1 demethylates methylated K482 and K615 in SMARCC1. Purified 1 μg of GST (spot 1) or GST-LSD1 proteins (spot 2) were incubated with 100 ng of monomethylated K482 (**c**) or monomethylated K615 (**d**) peptides for 4 h at 37 ^o^C and the resulting peptides and control input methylated peptides (spots 3 and 4) were spotted onto a nitrocellulose membrane. The methylated peptides were detected by affinity-purified anti-monomethylated K482 (**c**) or K615 (**d**) peptide antibodies (see Supplementary Fig 4c) as indicated. **e** HeLa cells were transfected with 50 nM siRNAs of luciferase or SET7 for 48 h and the K615-methylated SMARCC1, SMARCC1, and other proteins were analyzed by Western blotting analysis using indicated antibodies. Source data are provided as a Source Data file.

Our protein sequence examination revealed that SMARCC1 contains three lysine residues, Lys201 (K201), Lys482 (K482), and Lys615 (K615), whereas SMARCC2 also has three lysine residues, Lys328 (K328), Lys457 (K457), and Lys592 (K592), in the putative H3K4-like methylation consensus motifs (Fig. 3a)^25,26,28,41^. Among these lysine residues, the K482 or K615 motif in SMARCC1 is conserved (K482) or partially conserved (K615) in the K457 or K592 motif of SMARCC2, respectively. To test whether these putative methylated lysine residues in SMARCC1 serve as substrates for L3MBTL3 and LSD1, we synthesized monomethylated K482 and K615 peptides and their unmethylated cognate peptides derived from SMARCC1 (Fig. 3b). We found that in vitro translated and ^35^S-labeled L3MBTL3 protein selectively and directly binds to the methylated K482 and K615 peptides immobilized onto Sepharose beads^41^, but not to their cognate unmethylated peptide beads (Fig. 3b). We also examined whether LSD1 can remove the methyl groups from the synthetic monomethylated K482 and K615 peptides by mixing the methylated peptides with the purified recombinant GST-LSD1 fusion protein or GST control^25,41^. We found that GST-LSD1 can effectively demethylate the methylated K482 or K615 peptides (Figs. 3c, d, and Supplementary Fig. 4c). We next investigated the requirement of SET7 for SMARCC1 methylation in vivo^26,41^. While the affinity-purified anti-methylated K615 antibody selectively recognized a 155 kilodalton (kDa) protein corresponding to the size of SMARCC1 protein in the total HeLa cell lysate, silencing of SET7 greatly reduced the intensity of this protein, suggesting that it is the SMARCC1 protein methylated at K615 by SET7 (Fig. 3e). However, our affinity-purified anti-methylated K482 antibody produced a high background in cell lysates so we could not directly evaluate the methylated K482 levels in various cells.

**Fig. 4:**
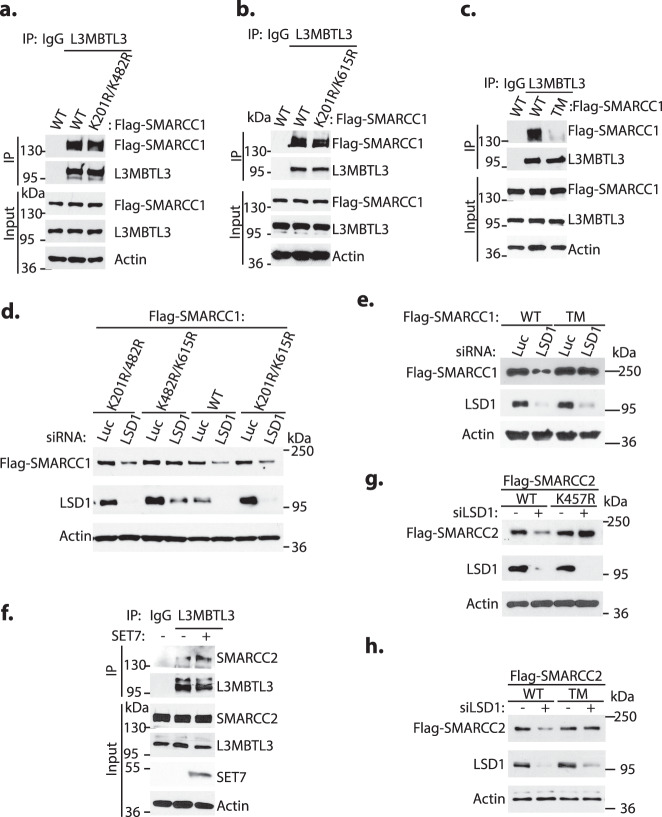
L3MBTL3 interacts with the methylated K482 and K615 to regulate SMARCC1. **a**–**c** K482 and K615 of SMARCC1 are required for interacting with L3MBTL3. The wildtype (WT), K201R/K482R (**a**) and K201R/K615R (**b**) double mutants, or K201R/K482R/K615R (**c**) triple mutants of Flag-SMARCC1 were transfected into 293 T cells for 48 h and cell lysates were immunoprecipitated by anti-L3MBTL3 antibodies or IgG (control). The interactions between Flag-SMARCC1 or its mutants and L3MBTL3 were detected by antibodies against indicated proteins. **d**, **e** Mutations of K482 and K615 confer resistance to LSD1 silencing. H1299 cells stably expressing the wildtype, K201R/K482R, K482/K615R, or K201R/K615R double mutants (**d**), or the K201R/K482R/K615R triple mutant of SMARCC1 (**e**) were transfected with 50 nM siRNAs of luciferase or LSD1 for 48 h and the levels of Flag-SMARCC1 protein and its mutants were analyzed by Western blotting. Repeated three times with similar results. **f** SET7 promotes interaction between L3MBTL3 and SMARCC2. H1299 cells stably expressing Flag-SMARCC2 were transfected with either the control empty vector or SET7 expressing vector for 48 h and cell lysates were immunoprecipitated by IgG control or anti-L3MBTL3 antibodies. The interaction between SMARCC2 and L3MBTL3 were analyzed. **g**, **h** The K457R and K328R/K457R/K592R mutants of SMARRC2 are resistant to LSD1 silencing. H1299 cells expressing the wildtype, K457R single (**g**) or K328R/K457R/K592R triple (**h**) mutant of SMARCC2 were transfected with 50 nM siRNAs of luciferase or LSD1 for 48 h and representation from three independent experiments is shown for the levels of Flag-SMARCC2 wildtype and mutants analyzed by Western blotting as indicated. Source data are provided as a Source Data file.

To define the critical lysine residues in SMARCC1, we mutated K201, K482, or K615 of SMARCC1 to arginine to produce K201R, K482R, and K615R mutants either singly or in combination so that they cannot be methylated by SET7 (Fig. 3a). Expression of K201R/K482R or K201R/K615R double mutants indicate that the presence of either K615 or K482 is sufficient for SMARCC1 to interact with L3MBTL3 (Fig. 4a, b). However, the K201R/K482R/K615R triple mutant (TM) of SMARCC1 failed to interact with L3MBTL3 (Fig. 4c). To further test if SET7 promotes the methylation of SMARCC1 in vivo, we co-transfected either the wild-type GFP-SMARCC1 or the GFP-SMARCC1 triple-mutant and SET7, and we found that only the wild-type SMARCC1, but not the SMARCC1 triple-mutant, was methylated by SET7 (Supplementary Fig. 4d). Consistently, while the K201R/K482R and K201R/K615R double mutants of SMARCC1 are still sensitive to LSD1 silencing, the K482R/K615R double mutant and in particular the K201R/K482R/K615R triple mutant are much more resistant to LSD1 silencing (Fig. 4d, e and Supplementary Fig. 5a, b). Furthermore, the K201R/K482R/K615R triple mutant is also resistant to be polyubiquitinated by SET7, L3MBTL3, and DCAF5 (Fig. 2h). Our results indicate that the methylated SMARCC1 protein is directly recognized by L3MBTL3 to promote its ubiquitin-dependent degradation by the CRL4^DCAF5^ ubiquitin E3 ligase and that LSD1 acts as a demethylase to remove the methyl groups from methyl-lysine 482 and methyl-lysine 615 of SMARCC1 to prevent the proteolysis of SMARCC1 by L3MBTL3 and the CRL4^DCAF5^ ubiquitin E3 ligase complex.

Since SMARCC2 also contains lysine residues in the putative H3K4-like methylation motifs (Fig. 3a), our studies revealed that the binding of SMARCC2 to L3MBTL3 is stimulated by SET7 (Fig. 4f). We also constructed the lysine-to-arginine mutations of either K457 (K457R) alone, which is identical to K482 in SMARCC1, or the triple mutations of K328R/K458R/K592R of SMARCC2 (Fig. 3a). We found that the K457R single and the K328R/K457R/K592R triple mutants (TM) of the Flag-tagged SMARCC2 are much more resistant to the loss of LSD1, as analyzed by direct Western blotting or by measuring the steady-state protein stability of SMARCC2 and its mutants after LSD1 silencing (Fig. 4g, h and Supplementary Figs. 5c, d, 6b,c). Our studies indicate that LSD1 and L3MBTL3 act through the H3K4-like lysine residues, such as K457 of SMARCC2, to regulate SMARCC2 protein stability.

### Regulation of SMARCC1 and mSWI/SNF complexes in embryonic stem cells

**Fig. 5:**
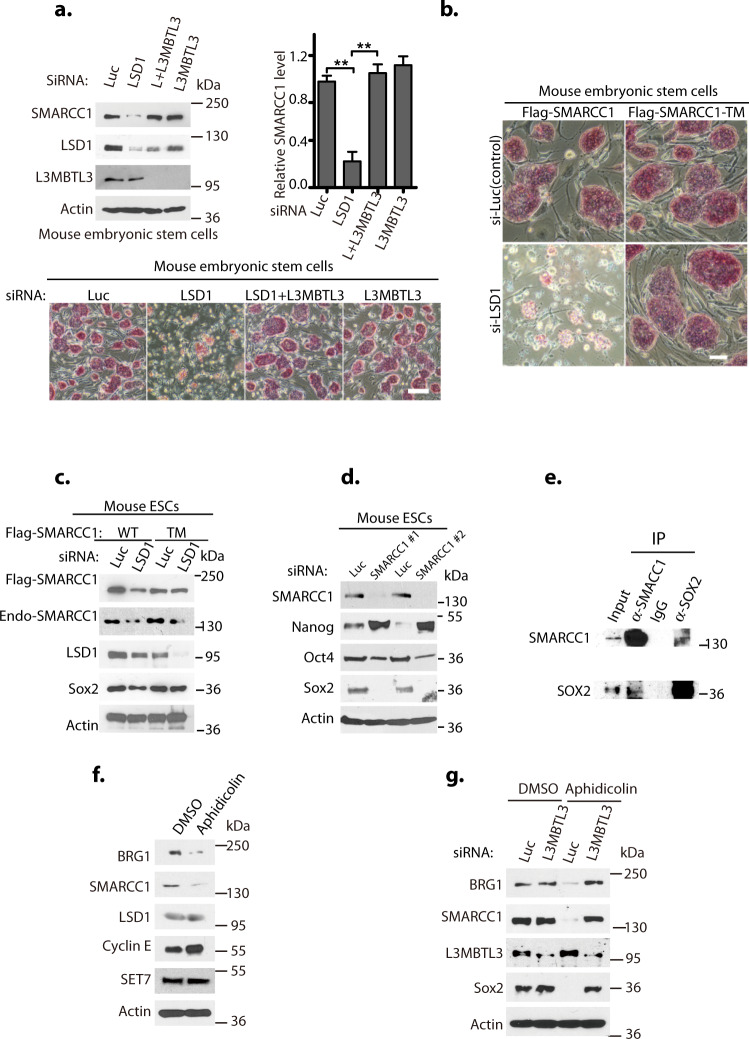
SMARCC1 is a critical target of LSD1 in mouse ESCs. **a** L3MBTL3 silencing stabilizes SMARCC1 in mouse embryonic stem cells. The mESCs were transfected with 50 nM of luciferase, LSD1, LSD1 + L3MBTL3, and L3MBTL3 siRNAs for 40 h. Representation from three independent experiments is shown for the levels of SMARCC1, LSD1, L3MBTL3, and actin examined by Western blotting and quantitatively analyzed. Significance was indicated as a two-tailed, unpaired, *t*-test for **a**. Values are expressed as the mean ± SEM. **p < 0.01. **b**, **c** The SMARCC1 triple mutant confers resistance to LSD1 silencing. The mESCs stably expressing the wildtype and the K201R/K482R/K615R triple mutant of Flag-SMARCC1 were transfected with 50 nM siRNAs of luciferase or LSD1 for 40 h, stained with alkaline phosphatase (AP), and cell images were acquired (**b**) with Nikon ECLIPSE Ti-S microscope equipped with NIS-Elements BR 3.1 software. Representation from three independent experiments for the levels of endogenous and ectopically expressed SMARCC1, LSD1, SOX2, and actin is shown by Western blotting with respective antibodies (**c**). **d** SMARCC1 silencing downregulates OCT4 and SOX2 and upregulates NANOG in mESCs. The mESCs were transfected with 50 nM of luciferase or two independent SMARCC1 siRNAs for 40 h. SMARCC1, OCT4, SOX2, NANOG, and actin were examined by Western blotting. **e** Endogenous SOX2 and SMARCC1 proteins were immunoprecipitated from mESCs and reciprocally blotted with respective antibodies. Input: 1/10 of cell lysates were used. **f** Aphidicolin causes downregulation of BRG1 and SMARCC1 in mESCs. The mESCs were treated with dimethyl sulfoxide (DMSO) or 5 μg/ml aphidicolin for 12 h and representation from two independent experiments shows the levels of BRG1, SMARCC1, LSD1, and cyclin E by Western blotting. **g** L3MBT3 silencing stabilizes BRG1 and SMARCC1 proteins in aphidicolin-treated mESCs. The mESCs were transfected with 50 nM of luciferase or L3MBTL3 siRNAs for 20 h and then treated with dimethyl sulfoxide (DMSO) or 5 μg/ml aphidicolin for another 12 h. Representation from three independent experiments is shown for the levels of BRG1, SMARCC1, LSD1, L3MBTL3, and SOX2 by Western blotting. Source data are provided as a Source Data file.

**Fig. 6:**
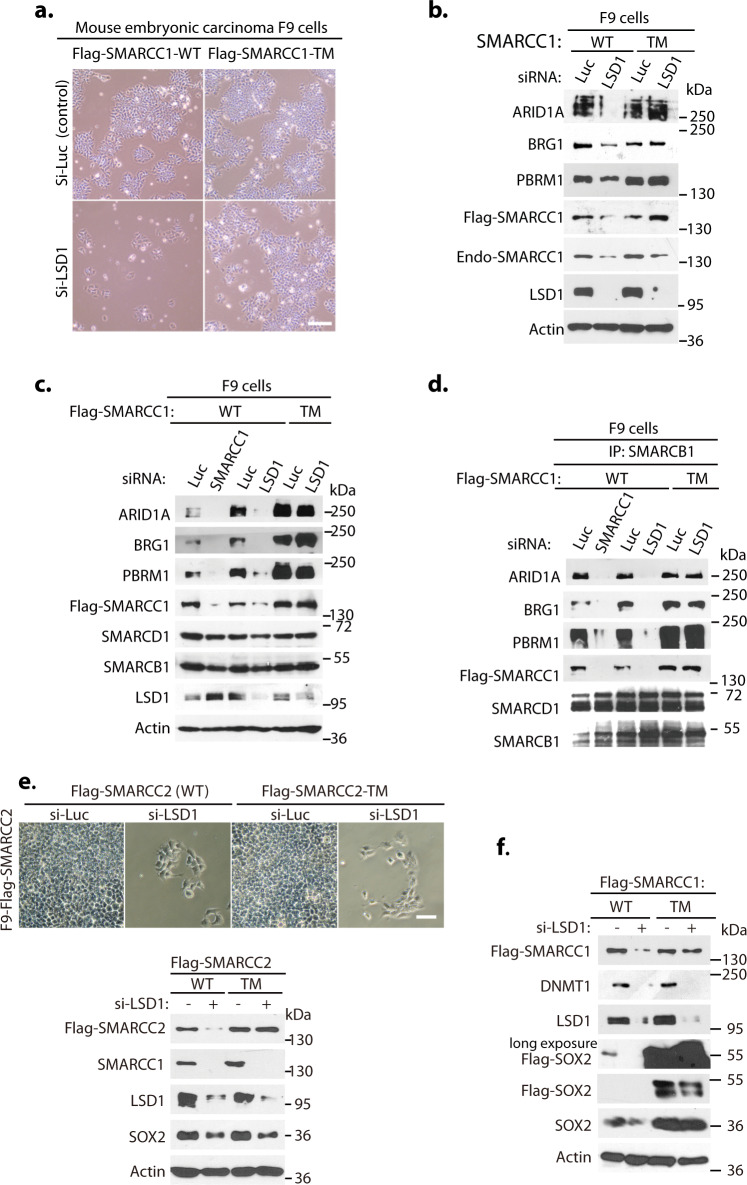
Mutations of SMARCC1 confer the resistance to LSD1 silencing in F9 embryonic carcinoma cells. **a**, **b** The wildtype and the K201R/K482R/K615R triple mutant of Flag-SMARCC1 were stably expressed in mouse F9 embryonic carcinoma cells. The cells were transfected with 50 nM siRNAs of luciferase or LSD1 for 40 h and cell images were acquired (**a**) and representation of three independent experiments is shown for mSWI/SNF proteins examined by Western blotting with respective antibodies (**b**). **c**, **d** F9 cells stably expressing the wildtype and the K201R/K482R/K615R triple mutant of Flag-SMARCC1 were transfected with 50 nM of luciferase, SMARCC1, or LSD1 siRNAs for 40 h as indicated. A set of lysates from a representation of three independent experiments is shown for direct Western blotting (**c**) and the other set for immunoprecipitation with anti-SMARCB1 antibodies, followed by Western blotting with antibodies for indicated SWI/SNF proteins (**d**). **e** Flag-SMARCC2 and its triple mutant in pMSCV-Puro were stably expressed in F9 cells. After transfecting luciferase or LSD1 siRNAs for 40 h, cell images were acquired with a Nikon ECLIPSE Ti-S microscope and indicated proteins were Western blotted. Two independent experiments were performed with the same results. **f** Flag-SOX2 in pMSCV-zeocin and Flag-SMARCC1 or its triple mutant in pMSCV-puromycin were co-expressed and selected in F9 cells. The stable cells were transfected with 50 nM luciferase or LSD1 siRNAs for 40 h, and Flag-SOX2, Flag-SMARCC1, and other indicated proteins were analyzed by Western blotting. Source data are provided as a Source Data file.

Mouse embryonic stem cells (mESCs) and mouse F9 embryonic carcinoma cells uniquely express a specific mSWI/SNF complex, the esBAF complex, that only contains SMARCC1 but not SMARCC2^4–7^ (Supplementary Fig. 7e). We found that siRNA-mediated silencing of LSD1 caused the proteolysis of SMARCC1 and greatly impaired the self-renewal and pluripotency of mESCs, indicated by the loss of alkaline phosphatase (AP) activity (Fig. 5a, b). The co-silencing of L3MBTL3 and LSD1 restored the SMARCC1 protein level and rescued the defects on self-renewal and pluripotency in LSD1 deficient mESCs (Fig. 5a), indicating that L3MBTL3 mediates the proteolysis of SMARCC1 in mESCs^26^. LSD1 silencing also blocked the proliferation of F9 cells, induced SMARCC1 proteolysis, and promoted the reduction of other mSWI/SNF proteins (Fig. 6a, b and Supplementary Fig. 7c, d). The effects of LSD1 silencing are unique for mESCs and F9 cells, as silencing of LSD1 in human embryonic kidney carcinoma 293 T and lung carcinoma H1299 cells that express both SMARCC1 and SMARCC2 did not exhibit any proliferation defects (Supplementary Fig. 7c–e).

While our studies and other reports indicate that LSD1 plays an essential role in both human and mouse ESCs^22,23,25,26^, the mechanism by which LSD1 regulates ESCs remains unclear. We found that SMARCC1 silencing using two representative siRNAs impairs the self-renewal of both mESCs and F9 cells but not the proliferation of 293 T or H1299 cells^4,5^ (Supplementary Figs. 7c–e, 8e), similar to the selective effects of LSD1 silencing in these cells. To analyze the role of SMARCC1 in mESCs and F9 cells, we ectopically and stably expressed Flag-SMARCC1 and its lysine-to-arginine mutants using the pMSCV-Puro retroviral LTR vector in both mESCs and F9 cells. Strikingly, we found that stable expression of the K201R/K482R/K615R triple mutant of Flag-SMARCC1, but not the wildtype counterpart, is sufficient to confer the resistance to LSD1 silencing to maintain the pluripotency and self-renewal in mESCs (Fig. 5b, c). Similarly, the expression of Flag-SMARCC1 triple mutant, as well as the K482R/K615R double mutant, in F9 cells are sufficient to render the resistance to LSD1 silencing to prevent the degradation of the mSWI/SNF proteins and growth arrest (Fig. 6a, b and Supplementary Fig. 7a, b). Thus, the effect of LSD1 silencing appears to resemble the loss of SMARCC1 in mESCs and F9 cells, since the loss of either LSD1 or SMARCC1 blocked cell proliferation, induced the disassembly of the mSWI/SNF complex, and the degradation of mSWI/SNF subunits, as analyzed by the immuno-co-precipitation of the mSWI/SNF complex by anti-SMARCB1 antibodies (Figs. 5b, c, 6a–d). We further investigated whether ectopic expression of SMARCC2 affects the sensitivity of LSD1 deficiency in these pluripotent cells. The Flag-SMARCC2 in the pMSCV-Puro retroviral vector was stably expressed in F9 cells and the response to LSD1 inactivation was analyzed. We found that the ectopic and stable expression of the Flag-SMARCC2 and its triple mutant cannot confer resistance to LSD1 deficiency-induced growth inhibition and SMARCC1 degradation (Fig. 6e). These studies suggest that SMARCC1 is a key target of LSD1 in both mESCs and F9 cells and SMARCC2 cannot substitute SMARCC1 for this critical function. Notably, our analysis revealed that the silencing of either LSD1 or SMARCC1 also induced the expression of endogenous SMARCC2 in F9 cells or mESCs (Supplementary Fig. 7d), suggesting that loss of SMARCC1/LSD1 and pluripotency might induce the expression of endogenous SMARCC2 in the LSD1- or SMARCC1-deficient pluripotent cells. In contrast, silencing of LSD1 or SMARCC1 caused the downregulation of SMARCC2 in both 293 T and H1299 cells (Supplementary Fig. 7d), indicating that the regulation of mSWI/SNF complex in mESCs and F9 cells is very distinct from that of 293 T and H1299 cells and that the expression of SMARCC2 alone may not be the only factor that affects the resistance of 293 T and H1299 cells to LSD1 silencing (Supplementary Fig. 7c–e).

The mSWI/SNF complex is essential for mESCs and F9 cells through their regulation of the central OCT4-SOX2-NANOG transcription circuitry^4–7^. Indeed, silencing of SMARCC1 caused the downregulation of OCT4 and SOX2, and the upregulation of NANOG proteins in mESCs (Fig. 5d). We have previously shown that loss of LSD1 causes the proteolysis of SOX2 protein through the L3MBTL3 and CRL4^DCAF5^ complex^25,26^. Notably, while LSD1 silencing reduced SOX2 levels in mESCs or F9 cells expressing the wildtype Flag-SMARCC1, expression of the K482R/K615R double or the triple mutant of Flag-SMARCC1 significantly reversed the downregulation of SOX2 after LSD1 silencing in mESCs and F9 cells (Fig. 5c and Supplementary Figs. 6a, 7a, b). We further found that SMARCC1 and SOX2 interact with each other in mESCs (Fig. 5e). We also ectopically and stably co-expressed Flag-SOX2 in the pMSCV-zeocin vector and Flag-SMARCC1 or its triple mutant in the pMSCV-puromycin vector in F9 cells (Fig. 6f). We found that in F9 cells expressing the Flag-SMARCC1 triple mutant, but not the wildtype Flag-SMARCC1, the protein levels of Flag-SOX2 or the endogenous SOX2 became highly increased, although both forms of SOX2 proteins remain sensitive towards the loss of LSD1 (Fig. 6f). Intriguingly, the effect of SMARCC1 triple mutant on SOX2 is selective, as DNMT1, another LSD1 demethylase substrate, remained to be downregulated in response to LSD1 silencing in cells expressing either Flag-SMARCC1 or Flag-SMARCC1 triple mutant (Fig. 6f). These results demonstrate that the SMARCC1 triple mutant may selectively stabilize SOX2 protein, suggesting that SMARCC1 is a critical target of LSD1 in regulating the assembly of mSWI/SNF complex and the SOX2 protein levels to maintain the self-renewal and pluripotency of mESCs and F9 cells.

### Cell cycle regulation of mSWI/SNF proteins by L3MBTL3

We repeatedly found that aphidicolin, a DNA polymerase inhibitor that arrests replicating cells at the G1/S border and in the S phase, reduced the steady-state levels of SMARCC1 and BRG1 in mESCs, as compared to asynchronously growing cells (Fig. 5f, g). The aphidicolin-arrested cells are associated with reduced levels of SOX2 and increased levels of cyclin E (Fig. 5f, g), a late G1 and early S phase cell cycle regulator, in mESCs^41^. Notably, in aphidicolin-arrested S phase mESCs, L3MBTL3 silencing is sufficient to restore the levels of SMARCC1, BRG1, and SOX2 proteins (Fig. 5g). To characterize potential cell cycle regulation of mSWI/SNF complexes, we synchronized HeLa cells at the G1/S border by the sequential thymidine/aphidicolin treatment^41^, and measured the levels of mSWI/SNF proteins after releasing the synchronized cells into S phase in fresh media without aphidicolin. Our studies revealed that the protein levels of SMARCC1, SMARCC2, BRG1, PBRM1, and ARID1A are relatively low in the synchronized G1/S and early S phase cells, and their levels become gradually increased as cells progress towards late S phase and/or G2 phase (Supplementary Fig. 9a, b). Since aphidicolin inhibits DNA polymerases, we also synchronized HeLa cells in mitosis by nocodazole, an inhibitor for the polymerization of microtubules. The mitotic cells were collected, washed, and released into fresh culture media without nocodazole to allow these mitotic synchronized cells to progress into G1, S, G2, and M phases^50^. We subsequently measured the levels of mSWI/SNF proteins at various times after mitotic release. Our studies showed that the protein levels of SMARCC1, SMARCC2, BRG1, PBRM1, and ARID1A are dynamically regulated in the cell cycle and these proteins were reduced to low levels in the S phase cells (Supplementary Fig. 10a, b). These observations are consistent with our previous findings that L3MBTL3-regulated DNMT1 degradation increases in the S phase and that SOX2 is a substrate of L3MBTL3^25,26,41^. Our findings are also consistent with previous observations that SMARCC2 protein is undetectable in the S and G2 phases of proliferating embryonic cortical progenitor cells during mouse cortex development between E10.5-E14.5^15^. Together, these observations suggest that the levels of mSWI/SNF proteins are regulated in the cell cycle and that L3MBTL3 plays a role in the regulation of mSWI/SNF proteins in the S phase of the cell cycle.

### Control of subunit deficiency-induced SMARCC1/SMARCC2 proteolysis

**Fig. 7:**
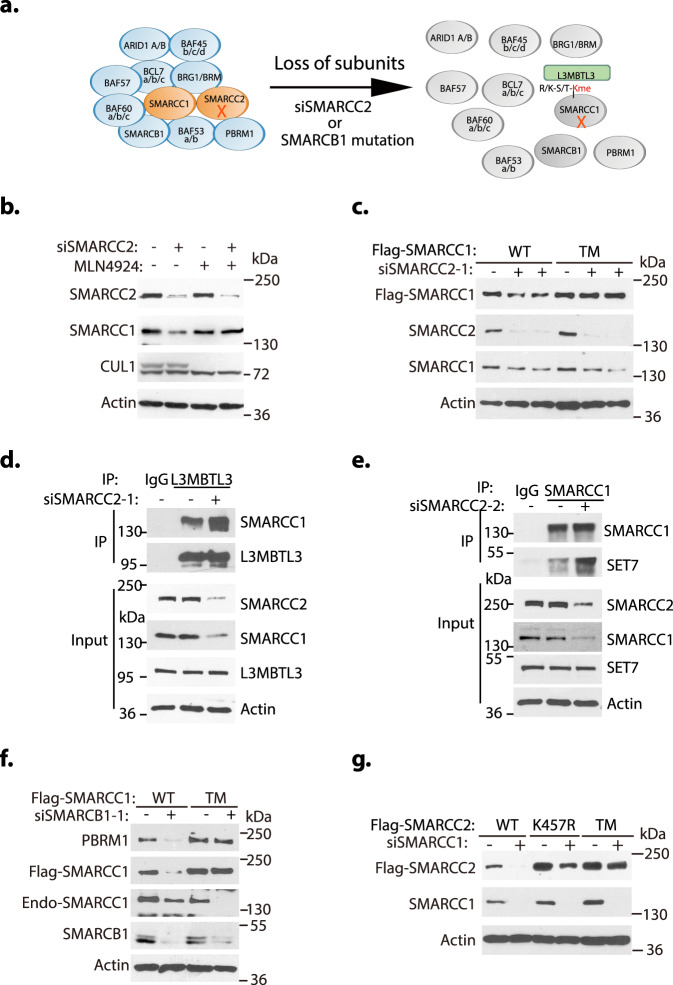
L3MBTL3 regulates the proteolysis of SMARCC1 and SMARCC2 during the disassembly of the SWI/SNF complexes. **a** Either loss or mutation of SWI/SNF components such as SMARCC2 or SMARCB1 leads to the proteolytic degradation of other subunits, including SMARCC1. **b** The proteolysis of SMARCC1 induced by SMARCC2 silencing is re-stabilized by MLN4924. HeLa cells were transfected with 50 nM siRNAs of luciferase or SMARCC2 for 42 h and the cells were treated with or without 2 μM MLN4924, a neddylation inhibitor, for the last 6 h as indicated. SMARCC1, SMARCC2, and CUL1 proteins were analyzed by Western blotting. **c** SMARCC1 triple mutant is resistant to SMARCC2 silencing. H1299 cells stably expressing wildtype or the K201R/K482R/K615R triple mutant of SMARCC1 were transfected with 50 nM siRNAs of luciferase or SMARCC2 for 48 h and representation of three independent experiments is shown for the levels of Flag-SMARCC1 and its mutant, endogenous SMARCC1, and SMARCC2 by Western blotting. **d**, **e** Loss of SMARCC2 enhances the interaction between SMARCC1 and L3MBTL3 or SET7. HeLa cells were transfected with 50 nM siRNAs of luciferase or SMARCC2 for 48 h and the interactions between SMARCC1 and L3MBTL3 (**d**) or SET7 (**e**) were examined by immunoprecipitation and Western blotting. **f** SMARCC1 triple mutant confers the resistance of SMARCB1 silencing. H1299 cells expressing wildtype or the K201R/K482R/K615R triple mutant of Flag-SMARCC1 were transfected with 50 nM siRNAs of luciferase or SMARCB1 siRNAs for 48 h and representation of three independent experiments is shown for Flag-SMARCC1 and the triple mutant, PBRM1, and actin proteins by Western blotting. **g** The SMARCC2 K457R single and K328R/K457R/K592R triple mutants are resistant to SMARCC1 silencing. H1299 cells expressing wildtype or the K328R/K457R/K592R triple mutant of SMARCC2 were transfected with 50 nM siRNAs of luciferase or SMARCC1 siRNAs for 48 h and a representation of two independent experiments on the levels Flag-SMARCC2 and its mutant proteins by Western blotting is shown. Source data are provided as a Source Data file.

It is well established that the subunit stoichiometry and composition of mSWI/SNF complexes are uniquely regulated that mutation/loss of a particular subunit, such as SMARCC1, SMARCC2, SMARCB1, BRG1, or ARID1A, destabilizes the mSWI/SNF complexes to induce proteolysis of other subunits via an unknown mechanism (Fig. 7a)^14,44,45,51^. Indeed, our studies showed that silencing of either SMARCC2 or SMARCB1 triggered the proteolysis of SMARCC1 and other mSWI/SNF proteins such as PBRM1 (Fig. 7b, c, f). We wondered whether L3MBTL3 and CRL4 are involved in SMARCC1 degradation during the mSWI/SNF disassembly induced by the loss of SMARCC2 or SMARCB1. Our tests revealed that SMARCC1 degradation, induced by SMARCC2 silencing, can be effectively reversed by MLN4924, a protein neddylation inhibitor that blocks the assembly of CRL ubiquitin ligase complexes including CRL4^52^ (Fig. 7b). Importantly, we repeatedly found that while silencing of SMARCC2 or SMARCB1 triggers the degradation of the wildtype Flag-SMARCC1 protein, the Flag-SMARCC1 triple mutant is resistant to the loss of either SMARCC2 or SMARCB1 (Fig. 7c, f and Supplementary Fig. 8a, c, d), and that the SMARCC1 triple mutant expression also stabilized other mSWI/SNF proteins such as PBRM1 (Fig. 7f). If SMARCC1 proteolysis during the silencing of SMARCC2 is mediated by L3MBTL3, we would expect that the interaction between SMARCC1 and L3MBTL3 to increase. Indeed, we found that SMARCC2 silencing enhanced the binding of SMARCC1 to L3MBTL3 (Fig. 7d) and silencing of SMARCC2 also enhanced the interaction between SMARCC1 and SET7 (Fig. 7e), suggesting that the released SMARCC1 after SMARCC2 silencing and subsequent disassembly of mSWI/SNF complexes may facilitate SMARCC1 methylation by SET7 and its subsequent binding to L3MBTL3. Conversely, while SMARCC1 silencing induces proteolysis of the wildtype Flag-SMARCC2^14,44,45^, the K457R and the triple mutants of Flag-SMARCC2 are more resistant to SMARCC1 silencing (Fig. 7g and Supplementary Fig. 8b). Thus, our results reveal that the proteolysis of SMARCC1 or SMARCC2 triggered by the loss of individual subunit of mSWI/SNF complexes is regulated by the L3MBTL3-CRL4^DCAF5^ dependent pathways.

### Regulation of SMARCC1/SMARCC2 by L3MBTL3 during development

**Fig. 8:**
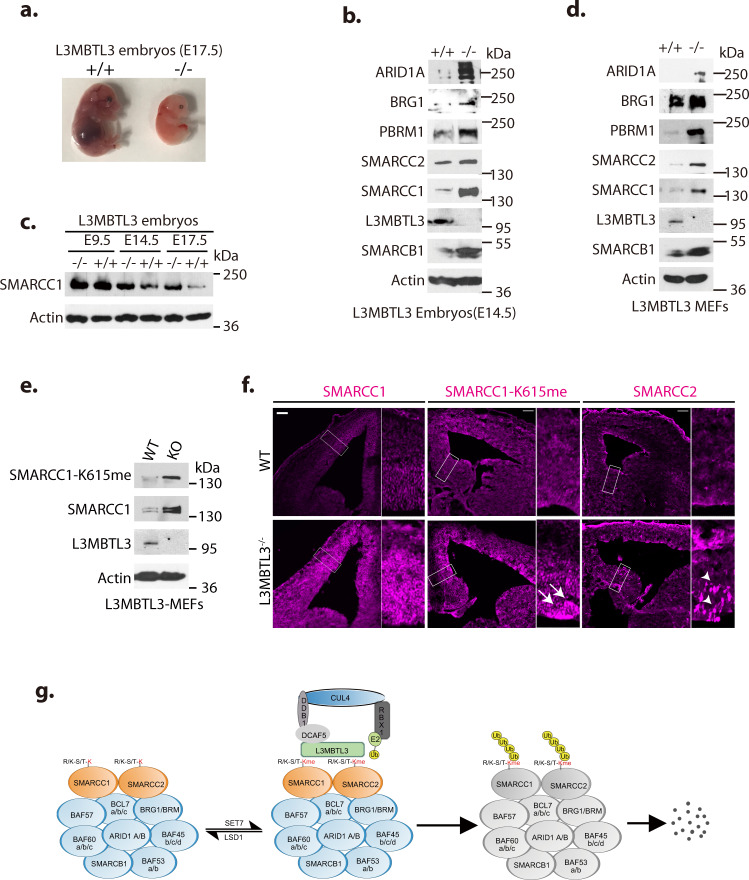
L3MBTL3 regulates the proteolysis of SMARCC1 and SMARCC2 during mouse development. **a** The mouse *L3MBTL3* wildtype (+/+) and *L3MBTL3* null (−/−, KO) mutant embryos on embryonic day 17.5 (E17.5) after breeding. The genotypes were determined from genomic DNA isolated from the wildtype and the *L3MBTL3* null embryos. Repeated three times with the same results. **b** Total lysates from the heads of mouse *L3mbt3* (+/+) and deletion (−/−) mutant embryos (equal total proteins) were analyzed by Western blotting with antibodies for the indicated mSWI/SNF proteins. **c** Lysates from the heads of wildtype and *L3mbtl3* deletion embryos were collected at various embryonic dates (E9.5, E14.5, and E17.5) and analyzed for SMARCC1 and actin protein levels. **d** Mouse embryonic fibroblasts from the wildtype and *L3mbtl3* deletion mutant embryos (E13.5) were examined and representation of three independent experiments is shown for indicated SWI/SNF proteins by Western blotting. **e** The *L3mbt3* (+/+) and deletion (−/−) mutant MEFs were analyzed for monomethylated K615 of SMARCC1, total SMARCC1, and L3MBTL3 proteins by Western blotting. **f** Embryonic brain cryo-sections of wildtype and *L3mbtl3* deletion mutant (E14.5, 10-µm thick coronal) were immunostained with anti-SMARCC1, monomethylated K615 of SMARCC1, and SMARCC2 antibodies. Boxed regions are enlarged and arrows or arrowheads indicate the increased SMARCC1-K615me or SMARCC2 regions in the brains. Scale bar: 100 μm. Repeated three times with similar results. **g** Model: Specific lysine residues of SMARCC1 and SMARCC2, core components of the SWI/SNF complex, are methylated by SET7 methyltransferase and the levels of methyl-lysines are reversibly removed by LSD1 demethylase. L3MBTL3 preferentially binds to the methyl-lysine residues of SMARCC1 or SMARCC2 to recruit the CRL4^DCAF5^ ubiquitin E3 ligase complex to target the methylated proteins for ubiquitin-dependent proteolysis, promoting the disassembly and proteolytic degradation of the mSWI/SNF complex. Source data are provided as a Source Data file.

Human *L3MBTL3* is mutated in medulloblastoma and is further implicated in other pathological disorders such as multiple sclerosis, insulin resistance, prostate cancer, and breast cancer^49,53–56^. Mouse homozygous *L3mbtl3* deletion is late embryonic lethal around E17.5^41,57^, although the molecular targets for the lethality remain unclear (Fig. 8a). We found that the protein levels of mSWI/SNF subunits, including SMARCC1, BRG1, SMARCC2, PBRM1, ARID1A, and SMARCB1 are markedly elevated in the head extracts of mouse *L3mbtl3* (−/−) deletion mutant embryos at E14.5, as compared to that of the wildtype littermates (Fig. 8b). Further characterization showed that the protein levels of SMARCC1 were high and comparable in both wildtype and *L3mbtl3* deletion mutant embryos at E9.5. In the wild-type embryos, SMARCC1 protein levels became gradually declined between E14.5-E17.5. However, the protein levels of SMARCC1 accumulated in the *L3mbtl3* deletion embryos during the E14.5-E17.5 developmental period and these *L3mbtl3* deficient embryos eventually died^41^ (Fig. 8a, c). These studies indicate that SMARCC1 protein is targeted for proteolysis by L3MBTL3 between E14.5-E17.5 during mouse embryonic development. In MEFs isolated from *L3mbtl3* homozygous deletion mutant embryos (Fig. 8d), mSWI/SNF proteins also accumulated and *L3mbtl3* deletion markedly increased the methylated K615 level in SMARCC1, as compared to the MEFs from the wildtype littermates (Fig. 8e). Immunostaining of brain tissues revealed that the protein levels of total and K615-methylated SMARCC1 and SMARCC2 proteins are significantly elevated in *L3mbtl3* deleted embryos, as compared to that of wildtype littermates (Fig. 8f). These studies indicate that L3MBTL3 regulates the proteolysis of SMARCC1 and SMARCC2 during embryonic brain development and that the accumulation of mSWI/SNF proteins may contribute to the embryonic lethality of mouse *L3mbt3* deletion mutants. Our studies are consistent with a model by which SMARCC1 and SMARCC2 proteins are methylated by SET7 to recruit L3MBTL3 and its associated CRL4^DCAF5^ ubiquitin ligase complex to target SMARCC1 and SMARCC2 proteins for degradation, thereby regulating the disassembly of the mSWI/SNF complex through the subsequent proteolysis of other mSWI/SNF subunits, and that LSD1 serves as a demethylase to remove the methyl groups from the methylated SMARCC1 and SMARCC2 proteins to prevent their degradation to preserve the integrity of the mSWI/SNF complexes (Fig. 8g).

## Discussion

In this report, we found that the assembly or disassembly of the mSWI/SNF chromatin remodeling complexes is dynamically regulated by a lysine methylation-dependent proteolytic mechanism involving the activities of the LSD1 demethylase and the L3MBTL3-CRL4^DCAF5^ ubiquitin ligase complexes. Our studies revealed that two key mSWI/SNF components, SMARCC1 and SMARCC2, contain critical lysine residues for SET7-mediated methylation and these methylated lysine residues serve as the substrates of LSD1 and L3MBTL3 that regulate the proteolysis of SMARCC1 and SMARCC2, promoting the disassembly of the mSWI/SNF complexes. Our studies are consistent with previous findings that SMARCC1 and SMARCC2 are the core mSWI/SNF components that are present in every mSWI/SNF complex for the nucleation of the mSWI/SNF complex assembly and that loss of SMARCC1 and SMARCC2 leads to the disassembly of entire mSWI/SNF complexes and the degradation of many mSWI/SNF subunits^15,43–45^. Notably, the loss of LSD1 blocked the self-renewal of mESCs and F9 cells that express the ESC-specific esBAF complex that contains only SMARCC1^4,5^, and our work identifies SMARCC1 as the critical target of LSD1 in mESCs and F9 cells (Figs. 5, 6). However, loss of LSD1 in other cells, such as 293 T or H1299 cells that contain both SMARCC1 and SMARCC2, has no detectable growth defects (Supplementary Fig. 7c–e), although the loss of LSD1 triggers the degradation of both SMARCC1 and SMARCC2 proteins in these cells. It is possible that the fractions of the methylated SMARCC1 and/or SMARCC2 proteins are not high enough to cover every mSWI/SNF complex in 293 T and H1299 cells for disassembly and degradation. Our studies also show that ectopic expression of SMARCC2 in F9 cells is not sufficient to confer the resistance to LSD1 inactivation (Fig. 6e), suggesting that additional mechanisms exist for 293 T and H1299 cells to resist LSD1 silencing. SMARCC1 or SMARCC2 are also proteolyzed when mSWI/SNF subunits are lost or mutated^14,44,45,51^. Our evidence suggests that the proteolysis of SMARCC1 and SMARCC2 during the loss of other mSWI/SNF subunits is L3MBTL3 dependent (Fig. 7). Although we found that SMARCC1 and SMARCC2 are regulated through lysine methylation, it remains to be further investigated whether additional mSWI/SNF proteins are similarly regulated by lysine methylation-dependent proteolysis through LSD1 and the L3MBTL3-CRL4^DCAF5^ ubiquitin ligase complexes.

## Methods

### Cells and cell synchronization

Human lung carcinoma H1299, cervical carcinoma HeLa, embryonic kidney carcinoma 293 T, and mouse teratoma F9 cells were purchased from the American Type Culture Collection (ATCC) and cultured in RPMI-1640 or DMEM medium with 10% FBS and 1% antibiotics as described^25,26,41^. Mouse embryonic fibroblasts (MEFs) were generated from wildtype and *L3mbtl3* deletion mutant embryos or CAGGCre-ER^TM^/LSD1^fl/fl^ embryos (E12.5-E13.5), as described previously^25,26,58^. Mouse embryonic stem cells (CMTI-2, strain C57/ BL6J, passage 16) were obtained from MilliporeSigma and were cultured on the mitomycin C treated mouse fibroblast feeder layer in knockout DMEM and knockout serum replacement, supplemented with leukemia inhibitory factor (LIF), GlutaMax, β-mercaptoethanol, MEM nonessential amino acid solution, and antibiotics^25,26^ (all from Life Technologies). For stable expression, human LSD1, SMARCC1, and SMARCC2 were cloned into the retroviral pMSCV-Puromycin or Zeocin vector containing 3xFlag-3xHA epitope (clontech) and the recombinant retroviruses were packaged in 293 T cells^25,26^. Viral-infected H1299, F9, and mouse embryonic stem cells were selected by puromycin or zeocin resistance. For cell cycle synchronization, actively growing HeLa cells were treated with 2.5 mM thymidine for 18 h, released into the fresh cell culture medium for 9 h, and then treated again with 5 μg/ml aphidicolin (Sigma) for another 15 h to synchronously arrest the cells at the G1/S border^41^. For cell cycle synchronization in mitosis, actively growing HeLa cells were treated with 40 ng/ml nocodazole for 14 h. The rounded-up mitotic cells were collected by low-speed centrifugation, washed, and released into fresh complete growth medium without nocodazole, and collected at various time points^50^. A fraction of cells at various time points after releasing from mitosis was labeled with 10 μM 5-ethynyl-2′-deoxyuridine (Edu) for 30 min, trypsinized, and processed according to the protocol of Clik-iTTM Plus Edu Flow Cytometry Assay Kit and stained by FxCycleTM PI/RNase Staining Solution at room temperature for 30 min^59^. Cellular DNA was stained with 4′,6-diamidino-2-phenylindole (DAPI).

### Peptide synthesis and preparation of methylated peptides

The monomethylated K482 (RALPEFFNGKNKS_(Km1)_TPEIYLAYRNFMIDTC) and K615 (GLRTDIYSKKTLAKS_(Kme1)_GASAGREWTEQETC) and cognate unmethylated peptides of SMARCC1 were synthesized at ABI Scientific. The monomethylated K482 and K615 peptides were used to raise rabbit polyclonal antibodies after covalently coupling these peptides to keyhole limpet hemocyanin (KLH)^41^. Affinity purification of methylated peptide antibodies were conducted as described in ref. 41, and the unmethylated and monomethylated K482 or K615 peptides were immobilized to Sulfolink-coupled-resins (Thermo Fisher) by covalently cross-linking with the cysteine residues at the end of the peptides to the resin^41^. The anti-monomethylated K482 or K615 peptide sera (5 ml each) were diluted in 1:1 in PBS and first passed through the unmethylated K482 or unmethylated K615 peptide columns (1 ml) three times to deplete anti-K482 or K615 peptide antibodies. The unbound flow-through antibody fractions were then loaded onto the monomethylated K482 or monomethylated K615 peptide column (0.5 ml), washed, and the bound antibody fractions were eluted by 5 ml of 100 mM glycine, pH2.5. The eluted antibodies (0.5 ml/fraction) were immediately neutralized by the addition of 100 μl of 2 M Tris, pH8.5, and tested for specificity towards the monomethylated K482 or K615 peptide but not to the unmethylated K482 or K615 peptide. For L3MBTL3 binding to methylated peptides, the monomethylated K482 and K615 peptides and cognate unmethylated peptides of SMARCC1 were covalently coupled to the Sulfolink-coupled resins, respectively, through the disulfide bond between the C-terminal cysteine of the peptides and the resins^25,26,41^. Human L3MBTL3 was cloned into pcDNA3 and in vitro translated and isotope-labeled using T7 polymerase TNT® Quick Coupled Transcription/Translation System (Promega) in the presence of ^35^*S*-l-methionine (PerkinElmer).

### RNA extraction and qRT-PCR analysis

RNA was extracted from the heads of mouse embryos or neonatal mice using Trizol regent according to the manufacturer’s instructions. 0.5 μg of total RNA was reverse-transcribed using a first-strand cDNA synthesis kit (Roche). qRT-PCR assays were performed with SYBR Green Master mix (Bio-Rad) and specific primers for PCR amplification. qRT-PCR data were recorded and analyzed using iQ-PCR (Bio-Rad) equipment and software according to manufacturers’ recommendations. For each primer pair, the primer efficiency was measured and the melting curve was analyzed. For each experiment, three technical replicates were used. The primers used for the qRT-PCR studies are: mouse SMARCC1 forward: ACAGGAGGAAAGGAAGATGAAG, reverse: GGATGCGTAGCTGGGAATAA; mouse SMARCC2 forward: AGTCAGGCAACCCTGTTATG, reverse: GGGCACTCTTCCTTCATTT; mouse LSD1 forward: TCAGTTTGTGCCACCTCTTC, reverse: GAACACACGGTCAAAGCATAAC; mouse BRG1 forward: TCACAGGCAAACTCCAGAAA, reverse: CCTCCTCATCTTCAGCCATAAG; mouse PBRM1 forward: AGGAGATGGGAGAAGAGGATAG, reverse: GTATAGGGCATGAGGTCCAAC; mouse SMARCB1 forward: TGGAGATTGCCATCCGAAATAC, reverse: TCATTCGCCTTGTGTTCCTATC; mouse ARID1A forward: GACCCAGGACAGAGAACATTAC, reverse: TCCTCTTCCTCCTCCAGTTTAG; mouse Actin forward: GTTACCAACTGGGACGACA and reverse: CCAGAGGCATACAGGGAC.

### Antibodies and immunological analysis

The commercially available antibodies used for Western blotting were anti-LSD1 (A300-215A, rabbit polyclonal, 1:2000), L3MBTL3 (A302-852, rabbit polyclonal, 1:1000), SMARCC2 (A301-038A, rabbit polyclonal, 1:1000), PBRM1 (A700-019, rabbit polyclonal, 1:1000), ARID1A (A301-040A, rabbit polyclonal, 1:1000), SMARCB1 (A301-087A rabbit polyclonal, 1:1000), BRG1 (A301-087A, rabbit polyclonal, 1:1000), BRM (A301-015A, rabbit polyclonal, 1:1000), and SET7 (A301-747A, rabbit polyclonal, 1:2000) antibodies purchased from Fortis Life Sciences; and the anti-BRG1 (49360, rabbit monoclonal, 1:1000), SMARCC1 (11956, rabbit monoclonal, 1:1000), HA (C29F4, rabbit monoclonal, 1:1000), and GFP (D5.1, rabbit monoclonal, 1:1000) antibodies were from Cell Signaling Technology. The anti-SMARCC1 (sc-32763, mouse monoclonal, 1:200) and β-actin (sc-47778, mouse monoclonal, 1:5000) antibodies were from Santa Cruz Biotechnologies. The anti-Flag (F1804, mouse monoclonal, 1:1000) antibodies were purchased from MilliporeSigma. We also used rabbit anti-L3MBTL3 and affinity-purified anti-DCAF5 antibodies we raised, both at 1:1000 dilutions, as described previously^41^. For direct Western blotting, cells were lysed in the 1XSDS sample buffer (4% SDS, 100 mM Tris, pH6.8, and 20% glycerol), quantified by protein assay dye (Bio-Rad), equalized by total proteins, and usually detected by specific antibodies at 1:1000-1:2000 dilutions^41^. For immunoprecipitation (IP), cells were lysed with an NP40-containing lysis buffer (0.5% NP40, 50 mM Tris, pH 7.5, 150 mM NaCl, and protease inhibitor cocktails)^41^. About 500 μg of lysates and 1 μg of antibodies were used for each immunoprecipitation assay. The antigen–antibody complexes were then pulled down by 30 μl Protein A Sepharose CL-4B (17-0963-03, GE Healthcare) and specific proteins were detected by Western blotting analysis, using specific antibodies at 1:1000 or 1:2000 dilutions and secondary goat anti-mouse HRP (Jackson Immuno Research, 115-035-008), goat anti-rabbit antibodies (Jackson Immuno Research, 111-035-008), or Protein A HRP (GE Healthcare, NA9120V), all at 1:5000 dilutions.

### Transfection and siRNAs

Oligofectamine was used for siRNA silencing in HeLa, H1299, or 293 T cells, and DharmaFECT1 was used for the siRNA silencing in mouse ESCs and F9 cells, whereas Lipofectamine 2000 was used for transient transfection as described previously^25,26,41^. Typically, 50 nM of each siRNA or their combinations were transfected into target cells for 48 h and cells were directly lysed in SDS or NP40 lysis buffers^41^. For verification of the effects, usually, two or three independent siRNAs were designed to examine the knockdown efficiency and the consequences of knockdown on target proteins^41^. The siRNAs for human genes are: LSD1-1: GGAAGAAGAUAGUGAAAAC; LSD1-2: AGUGAAAACUCAGGAAGAA; LSD1-3’UTR: GGGAGGAACUUGUCCAUUA; DCAF5-1: GCUGCAGAAACCUCUACAA; DCAF5-2: AUCACCAACUUCUGACAUA; L3MBTL3-1: GAUGCAGAUUCUCCUGAUA; L3MBTL3-2: GGUACCAACUGCUCAAGAA; SMARCC1: GGAACAAAGUGUCGGAACA; SMARCC1-2: GGAUGAAUGAGGAGGAUUA; SMARCC2-1: GCACAGACAUGUACACAAA; SMARCC2-2: GGAUGAGGAGAAAGG-GAAA; SMARCB1-1: ACGCUGAGAUGGAGAAGAA; SMARCB1-2: ACACUAAGGAUC-ACGGAUA; SET7-1: GGGCAGUAUAAAGAUAACA; SET7-2: ON-TARGETplus Human SETD7 (80854) siRNA-SMARTpool. The siRNAs for mouse genes are: mLSD1-2: AAGGA-AAGCTAGAAGAAAA; mL3MBTL3-1: GCTGAGGTTTGTGGATATA; mL3MBTL3-2: GCUCGAGGCUGCAGACAAA; mSMARCC1-1: GGAUGAAUGAAGAGGAUUA; mSMARCC1-2: GCUAACAAGUUGAAGAUA; mouse LSD1-1 is the same as human LSD1-1, and control siRNA for luciferase: CATTCTATCCTCTAGAGGA. All siRNAs were synthesized from Horizon Discovery.

### Demethylation analysis

The glutathione-*S*-transferase (GST) and the GST-LSD1 (human) fusion protein were expressed in *E. coli* BL21 strain and purified by the Glutathione Sepharose resin. Purified 1 μg of control GST or GST-LSD1 proteins were incubated with 100 ng of the monomethylated K482 or monomethylated K615 peptides for 4 h at room temperature and the resulting peptides were blotted onto nitrocellulose membrane^25,41^. The demethylated peptides were detected by immunoblotting with affinity-purified anti-monomethylated K482 or anti-monomethylated K615 antibodies.

### Animals and histology

The LSD1fl/ + conditional mutant (B6.129-Kdm1a tm1.1Sho/J, stock No: 023969), transgenic actin-Cre-ER (CAGGCre-ER^TM^, B6.Cg-Tg(CAG cre/Esr1*)5Amc/J, stock No: 004682), and transgenic Nestin-Cre (B6.Cg-Tg(Nes-cre)1Kln/J, stock No: 003771) mouse strains were obtained from Jackson Laboratory. The *L3mbt3* deletion mutant (*MBT-1*-/+, B6;129-L3mbtl3tm1Tmiy) mouse strain was previously described^41^. All animal experiments, including breeding, housing, genotyping, and sample collection were conducted in accordance with the animal protocols approved by the Institutional Animal Use and Care Committee (IACUC) and complied with all relevant ethical regulations at the University of Nevada, Las Vegas. All procedures were conducted according to the National Institutes of Health (NIH) Guide for Care and Use of Laboratory Animals. The UNLV IACUC is an AAALAC-approved facility and meets the NIH Guide for the Care and Use of Animals. For embryonic analyses, usually, three pairs of the *L3MBTL3* (−/+) male and female mice (10–12 weeks old) in three cages, each with one male and one female, were bred in the late afternoon and the breeding plugs were examined in the female mice in next morning. The positive plugs were counted as embryonic day 1 (E1) and the pregnant female mice between E14-E17.5 were euthanized by the primary method of CO_2_ asphyxiation, followed by cervical dislocation (secondary method), as approved by the institutional IACUC committee. Usually, a single pregnant female mouse produced about 6-8 embryos, which segregated at the Mendelian ratio, usually with 1–2 *L3MBTL3* (−/−), 1–2 wildtype, and 3–4 heterozygous *L3MBTL3* (−/+) embryos. The *L3MBTL3* null embryos between E17.5-19.5 usually died and became disintegrated, so they were excluded from protein analyses. For the analysis of mSWI/SNF proteins in LSD1^fl/fl^/Nestin-Cre mice, usually, three to four pairs of the LSD1^flox/flox^ male and LSD1^flox/+^/Nestin-Cre mice female mice (10–12 weeks old) were bred. The animals were collected immediately after birth to avoid any delay in sample analysis. The brains or other body parts of the mice were dissected for protein or immunostaining analysis. For immunostaining, embryos or dissected brains were fixed in 4% paraformaldehyde (PFA) at 4 °C overnight and embedded in an Optimal cutting temperature compound (OCT) according to standard procedures^27^. Sections (10-µm thick, coronal) were stained with specific antibodies and counter-stained with 4′,6-diamidino-2-phenylindole (DAPI). Images were acquired with the Nikon A1Rsi Confocal LSM. The sample size was chosen on the basis of our experience on *L3mbtl3* or *Lsd1* mutant mice and on cultured cells in order to detect the mSWI/SNF proteins for differences of at least 50% between the wildtype and mutant groups^41^. In the experimental analyses for the examination of proteins, the investigators were unaware of the genotypes of the experimental embryos. The investigators also randomly analyzed the wildtype, heterozygous and homozygous knockdown embryos.

### Analysis of proteins and DNA from embryos

The experimental procedures for embryo isolation were approved by the UNLV Institutional Animal Use and Care Committee (IACUC). The *L3mbtl3* embryos from the euthanized pregnant female mice or dissected brains or other body parts from the *Lsd1* mice were washed with PBS and lysed in the NP40 lysis buffer^41^. The nuclear and cytosolic fractions were separated by centrifugation. Genomic DNA was isolated from nuclear pellets by Zymo genomic DNA-tissue prep kit and quantified. Proteins in the cytosolic supernatant of the lysates were quantified by protein assay dye (Bio-Rad), equalized, and boiled for 15 min after the addition of 1% SDS and 5% beta-mercaptoethanol to the lysates. Proteins were resolved in protein gel and analyzed by Western blotting.

### Statistical information

Experiments were usually performed with at least two to three independent repeats to ensure the results. Statistical plot analyses were performed using Microsoft Excel. Protein bands were quantified using ImageJ. To quantify protein loading in each Western blot analysis of a set of protein samples, the same protein samples were analyzed with three repeated loading experiments (technical replicates). For cell-based assays, triplicated repeats in the same set of cells (technical replicates) were measured and the experiments were usually repeated in three independent experiments with independently cultured cells (biological replicates). Quantitative data are expressed by a bar graph, with mean and standard deviation (SD) for error bars from independent replicates. For siRNA-mediated knockdown experiments, statistically significant differences or variations between means of double and single knockdowns were normalized to the luciferase siRNA control or actin control and compared using a two-tailed paired Student’s *t*-test. For animal experiments, triplicated breeding was used to obtain a statistically significant number of embryos; and statistically significant differences between means of protein levels in the control wildtype and knockout mutants were compared using the two-tailed equal-variance independent Student’s t-test. All other data were determined using a two-tailed equal-variance independent Student’s *t*-test. The data in all figures met the assumption of normal distribution for tests. Different data sets were considered to be statistically significant when the *P* value was <0.05 (*) or 0.01 (**)^41,60^.

### Reporting summary

Further information on research design is available in the Nature Research Reporting Summary linked to this article.

## Supplementary information

Supplementary Information

Reporting Summary

## Data Availability

The data that support this study are available from the corresponding author upon reasonable request. Source data are provided with this paper.

## Source data

Source Data
