# Supplementary material for: The assembly of mammalian SWI/SNF chromatin remodeling complexes is regulated by lysine-methylation dependent proteolysis
Source: Nat Commun. 2022 Nov 5;13:6696. doi: 10.1038/s41467-022-34348-9 (PMC9637158; doi:10.1038/s41467-022-34348-9)
Supplement: Supplementary file 1 — Supplementary Information [file 41467_2022_34348_MOESM1_ESM.pdf]

The Assembly of Mammalian SWI/SNF Chromatin Remodeling Complexes is Regulated by Lysine-Methylation Dependent Proteolysis

Supplementary Information

Guo et al.

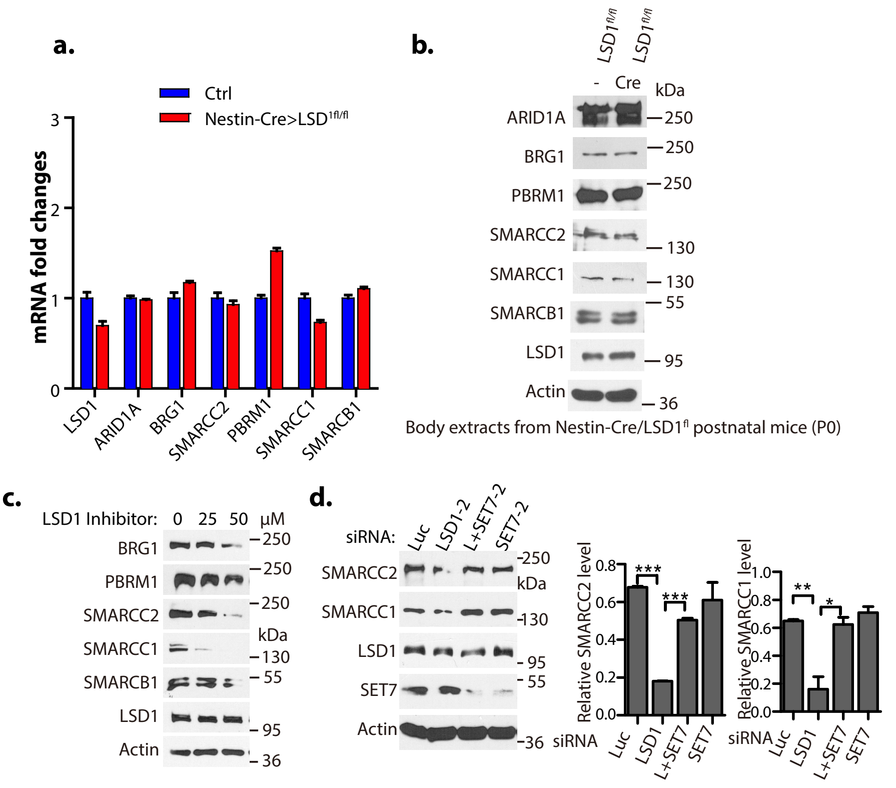

Supplementary Figure 1. Regulation of the mSWI/SNF complexes by LSD1. **a.** Reverse transcriptional quantitative PCR (RT-qPCR) analysis of the mRNA levels of mSWI/SNF components in the brain of the LSD1$^{flox/flox}$/Nestin-Cre mice. The mRNA levels were measured in triplicated by RT-qPCR. The quantifications are represented by bar graph with mean and standard deviation (S.D.) for error bars from three replicate samples and normalized to the control wildtype LSD1$^{flox/flox}$ mice. **b.** Western blotting analysis shows the indicated mSWI/SNF proteins remain unchanged in the extracts from the body part of LSD1$^{flox/flox}$/Nestin-Cre mice. **c.** Embryonic fibroblasts from the CAGGCre-ER^{TM}/LSD1$^{fl/fl}$ mouse embryos (E13.5) were treated with dimethyl sulfoxide (DMSO, 1%), and 25 □M or 50 □M of LSD1 inhibitor CBB3001 for 10 hours and the levels of mSWI/SNF proteins and LSD1 were analyzed by Western blotting. **d.** SET7 silencing stabilizes SMARCC1 and SMARCC2 proteins. HeLa cells were transfected with 50 nM indicated siRNAs of luciferase (Luc), LSD1, LSD1+SET7 (L+SET7), or SET7 for 48 hours and indicated proteins were analyzed by Western blotting. Band intensities were quantified by ImageJ software and normalized to the Luciferase (Luc) siRNA control signal and Significance was indicated as two-tailed, unpaired, *t* test. Values are expressed as mean ± SEM. *$p<0.05$, **$p<0.01$, ***$p<0.001$.

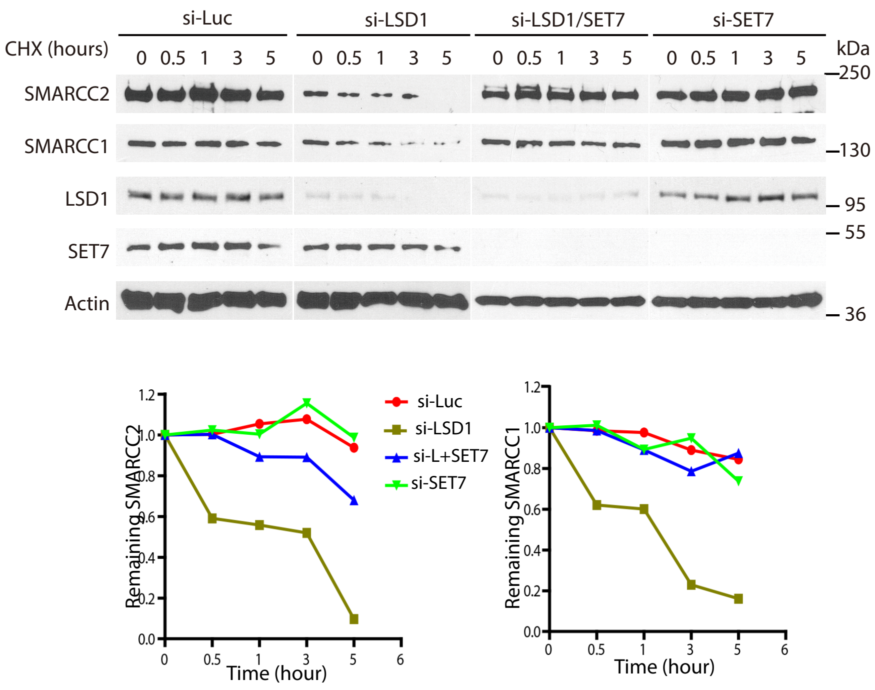

Supplementary Figure 2. HeLa cells were transfected with 50 nM siRNAs of luciferase (Luc), LSD1, LSD1+SET7, or SET7 for 45 hours. The cells were then treated with 100 μM cycloheximide (CHX) and collected at the indicated times to measure the half-lives of SMARCC1 and SMARCC2 proteins by Western blotting. The protein intensities were quantified and normalized to the intensity of SMARCC1 and SMARCC2 proteins at the zero time when cycloheximide was added.

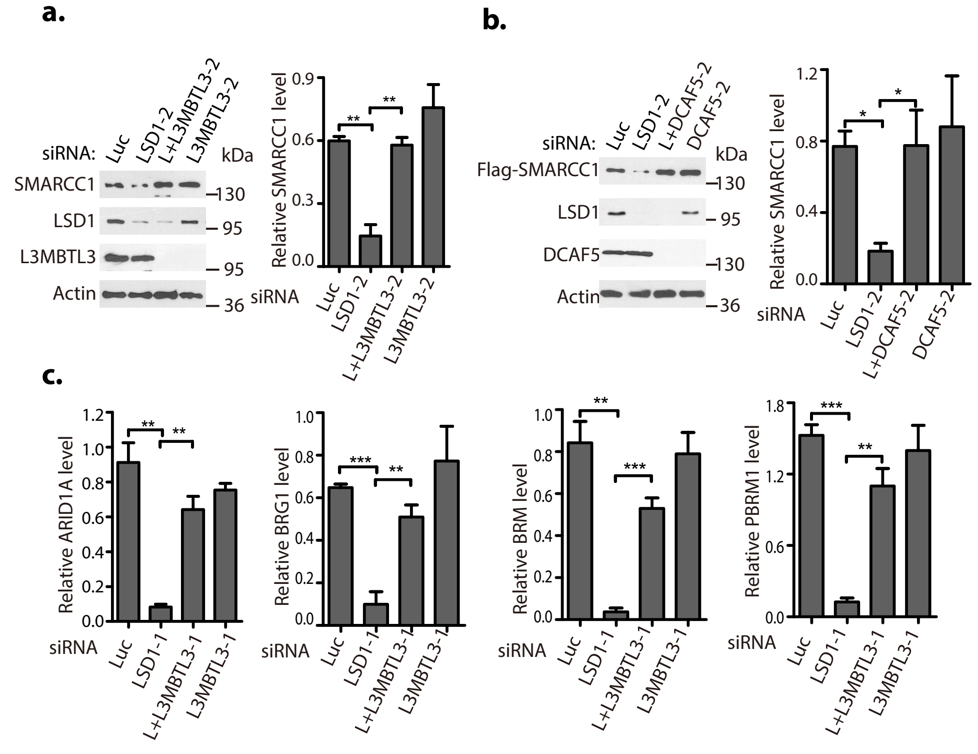

Supplementary Figure 3. Regulation of SWI/SNF complex by L3MBTL3 and DCAF5. **a.** Loss of L3MBTL3 stabilizes SMARCC1 in LSD1 deficient cells. HeLa cells were transfected with 50 nM indicated siRNAs of luciferase, LSD1, LSD1+L3MBTL3, or L3MBTL3 for 48 hours. SMARCC1 and other indicated proteins were analyzed by Western blotting. Band intensities were quantified and normalized to the Luciferase (Luc) siRNA control signal. **b.** Loss of DCAF5 stabilizes Flag-SMARCC1 in LSD1 deficient cells. The Flag-SMARCC1 in pMSCV-Puro was stably expressed in H1299 cells. The cells were transfected with 50 nM indicated siRNAs of luciferase, LSD1, LSD1+DCAF5, or DCAF5 for 48 hours and the indicated proteins were analyzed by Western blotting and quantified. **c.** The same as Fig. 2c and the protein band intensities in Fig. 2c were quantified and normalized to the Luciferase (Luc) siRNA control signal. Significance was indicated as two-tailed, unpaired, *t* test for (a-d). Values are expressed as mean ± SEM. *$p<0.05$, **$p<0.01$.

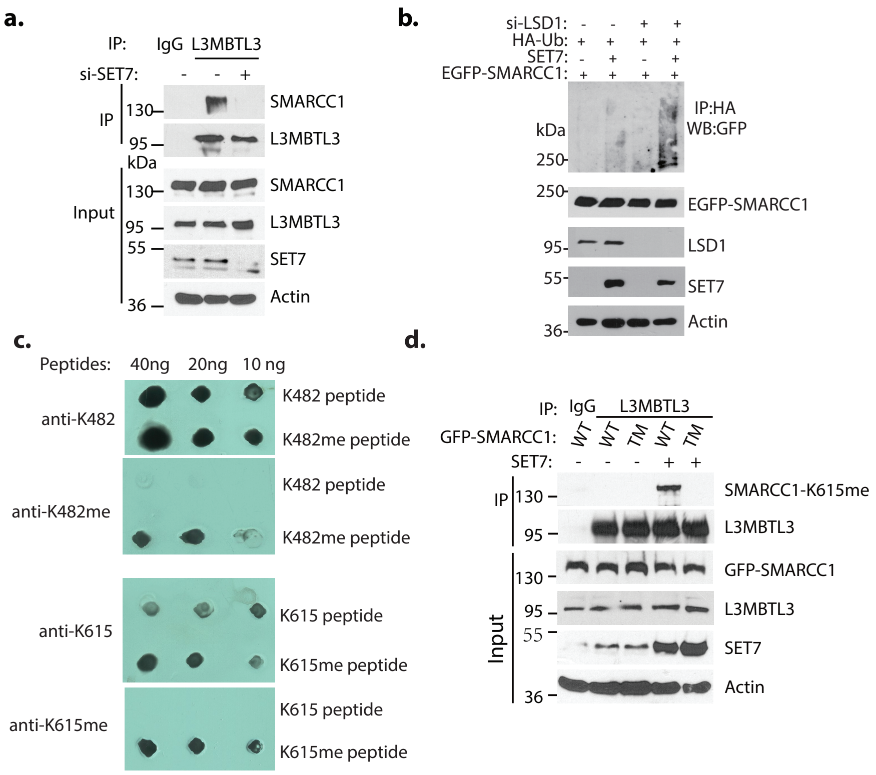

Supplementary Figure 4. a. Downregulation of SET7 reduces the interaction between SMARCC1 and L3MBTL3. HeLa cells were transfected with luciferase control and SET7 siRNAs and the interaction between SMARCC1 and L3MBTL3 was analyzed by the anti-L3MBTL3 immunoprecipitation, followed by Western blotting. **b.** 293T cells were first transfected with 50 nM Luciferase or LSD1 siRNAs for 12 hours. The EGFP-tagged SMARCC1 expressing vector was then co-transfected, together with vectors expressing HA-tagged ubiquitin (HA-Ub) in the presence or absence of SET7 expressing constructs as indicated. Proteins were immunoprecipitated with anti-HA antibodies and Western blotted with the anti-GFP-SMARCC1 and other indicated antibodies. **c.** The unmethylated and methylated K482 and K615 peptides were spotted onto nitrocellulose membrane in two sets. The top set was blotted by affinity purified anti-K482 or anti-K615 peptide antibodies and the bottom set was blotted by affinity purified anti-mono-methylated K482 or anti-mono-methylated K615 antibodies as indicated. **d.** The GFP-SMARCC1 and its triple mutants and SET7 expression constructs were co-transfected into 293T cells for 48 hours and cell lysates were immunoprecipitated by anti-L3MBTL3 antibodies or IgG (control). The interactions between GFP-SMARCC1-K615me and L3MBTL3 were detected by the affinity purified anti- K615me antibody and other antibodies against indicated proteins.

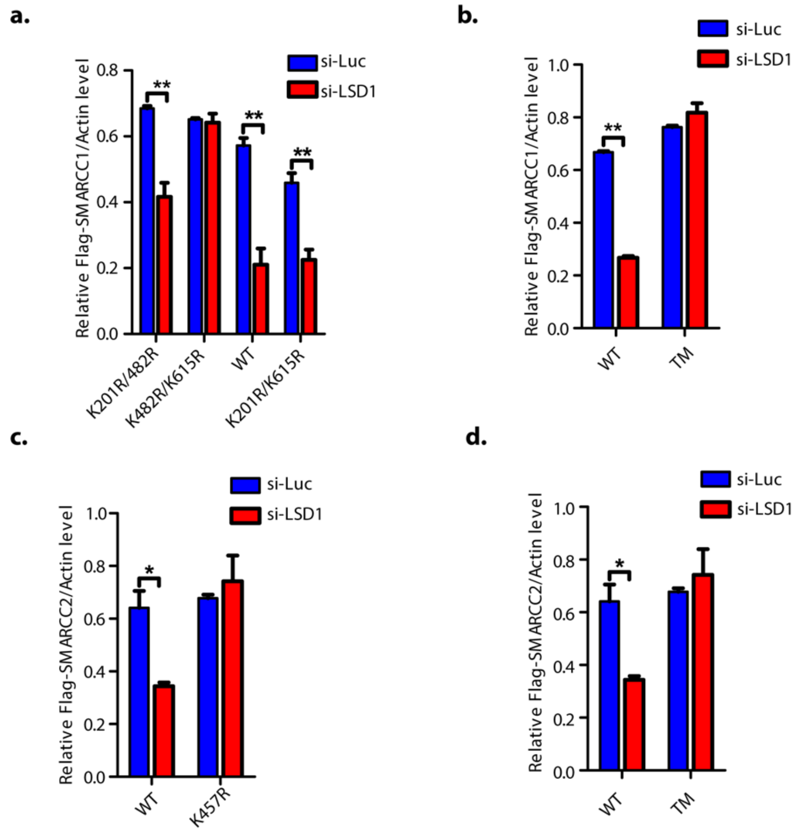

Supplementary Figure 5. a. The intensities of protein bands in the Western blots of Fig. 4d were quantified and normalized to the actin control signals. **b.** The intensities of protein bands in the Western blots of Fig. 4e were quantified and normalized to the actin control. **c.** The intensities of protein bands in the Western blots of Fig. 4g were quantified and normalized to the actin control. **d.** The intensities of protein bands in the Western blots of Fig. 4h were quantified and normalized to the actin control signals. Significance was indicated as two-tailed, unpaired, *t* test. Values are expressed as mean ± SEM. *$p<0.05$, **$p<0.01$.

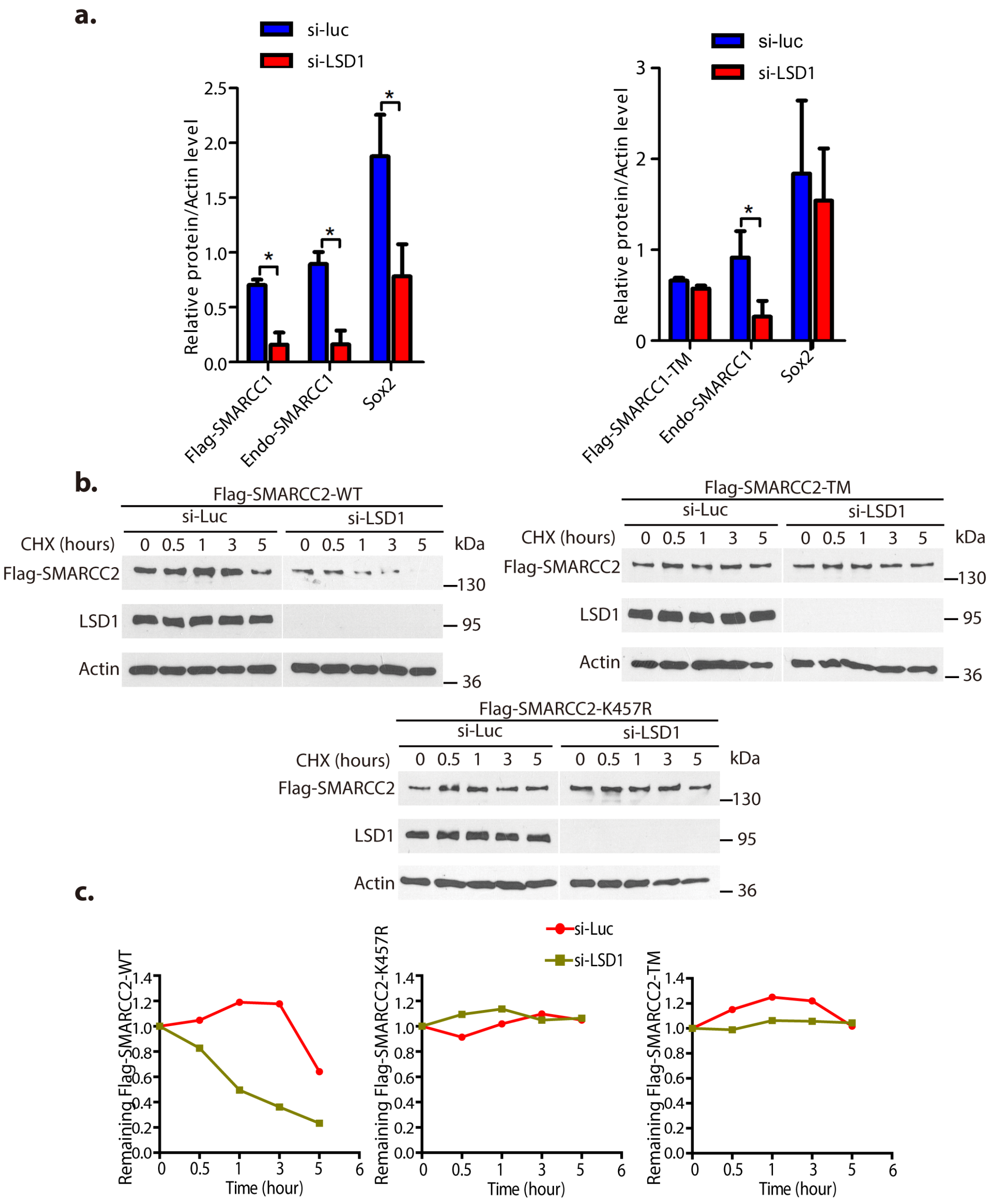

Supplementary Figure 6. a. The intensities of protein bands in the Western blots of Fig. 5c in mouse embryonic stem cells expressing Flag-SMARCC1 wildtype (left) and Flag-SMARCC1 triple mutant (right) were quantified and normalized to the actin control signals**.** The Flag-SMARCC2 wildtype (WT), K457R, and K328R/K457R/K592R triple mutants (TM) in pMSCV-Puro were stably expressed in H1299 cells. The cells were transfected with siRNAs of luciferase and LSD1 for 45 hours. The cells were then treated with 100 µM cycloheximide (CHX) and collected at the indicated times to measure the half-lives of SMARCC2 protein by Western blotting (**b**). The protein intensities were quantified by ImageJ software and normalized to the intensity of SMARCC2 at the zero time when cycloheximide was added (**c**). Significance was indicated as two-tailed, unpaired, *t* test. Values are expressed as mean ± SEM. *$p<0.05$.

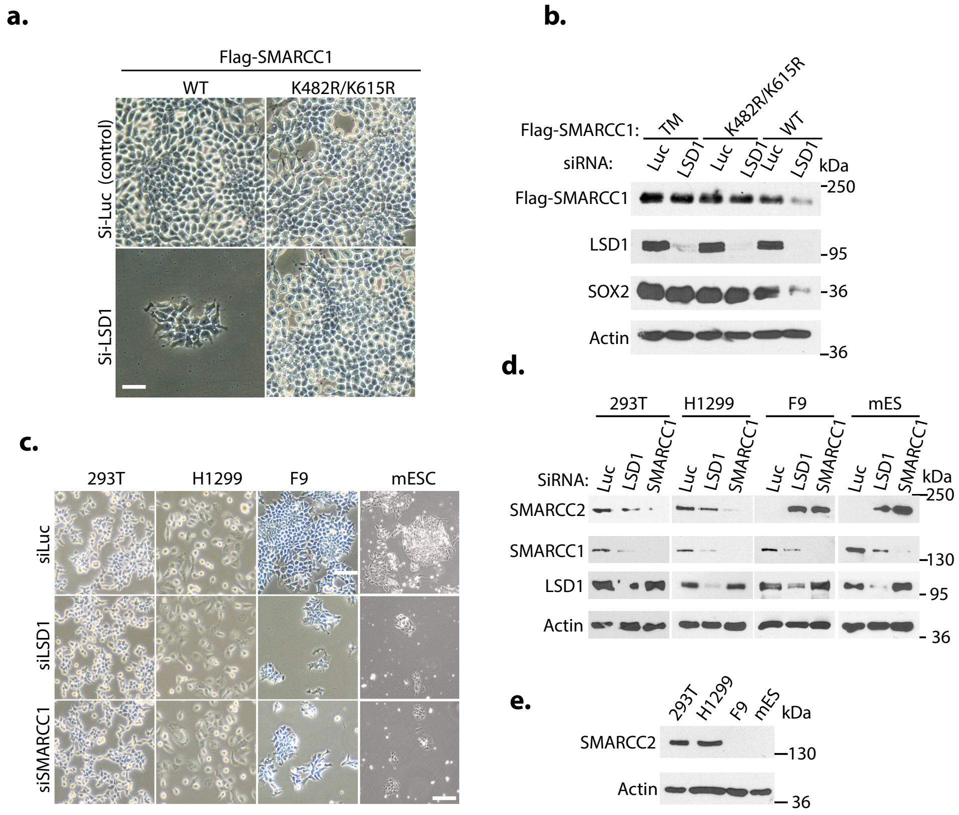

Supplementary Figure 7. a and **b.** F9 cells stably expressing the wildtype and the K482R/K615R double mutant of SMARCC1 were transfected with 50 nM of luciferase and LSD1 siRNAs for 40 hours and cell images were acquired (**a**) and Flag-SMARCC1, SMARCC1 mutant, LSD1 and SOX2 proteins were determined by Western blotting (**b**). **c-e.** 293T, H1299, F9 cells or mESCs were transfected with 50 nM of luciferase, LSD1 or SMARCC1 siRNAs for 40-48 hours and cell images were acquired (**c**) with Nikon ECLIPSE Ti-S microscope equipped with NIS-Elements BR 3.1 software and the cell lysates were blotted with anti-SMARCC1, SMARCC2, LSD1 and actin (loading control) antibodies (**d**). SMARCC2 is expressed in 293T and H1299 cells but is not detectable in F9 cells and mESCs (**e**). Scale bar: 200 µm.

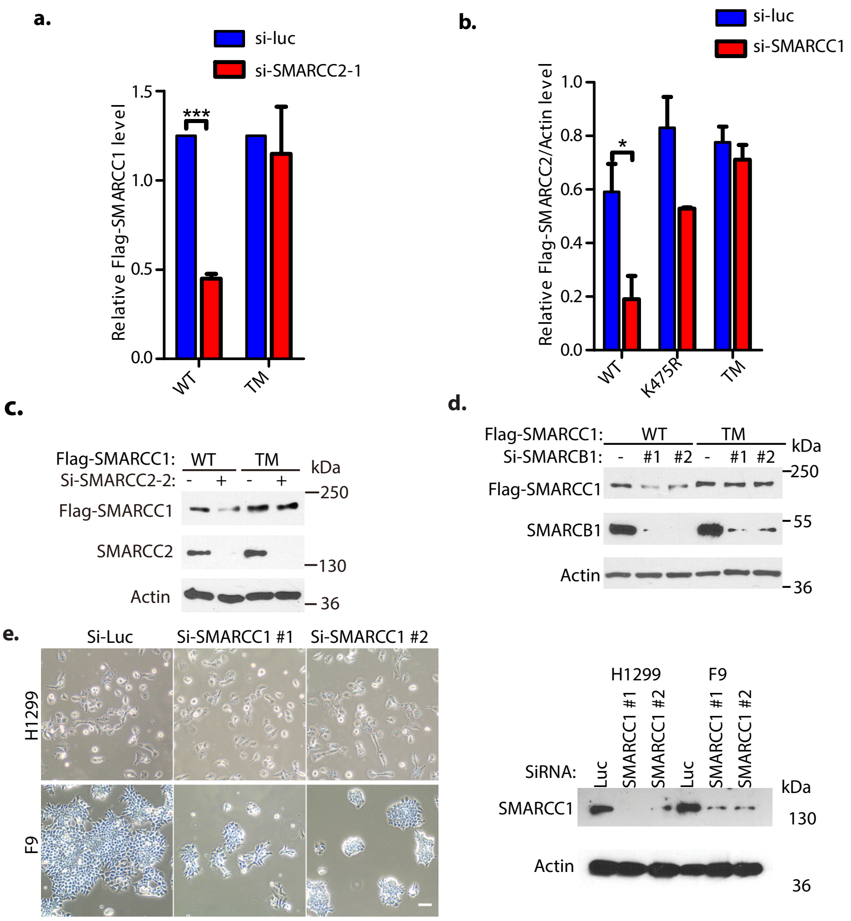

Supplementary Figure 8. a. The intensities of protein bands in the Western blot of Fig. 7c were quantified and normalized to the Luciferase (Luc) siRNA control. **b.** The intensities of protein bands in the Western blot of Fig. 7g and two repeats (total n=3) were quantified and normalized to the Luciferase (Luc) siRNA control **c.** The Flag-SMARCC1 and its K201R/K482R/K615R triple mutant in pMSCV-Puro were stably expressed in H1299 cells. The cells were transfected with 50 nM siRNAs of luciferase or SMACC2 for 48 hours, and Flag-SMARCC1, the triple mutant, SMARCC2, and actin proteins were analyzed by Western blotting. **d.** H1299 cells stably expressing the wildtype and the triple mutant of Flag-SMARCC1 were transfected with 50 nM of luciferase siRNA and two SMARCB1 siRNAs for 48 hours. SMARCC1, SMARCB1, and actin were analyzed by Western blotting with respective antibodies. **e.** H1299 or F9 cells were transfected with 50 nM of luciferase siRNA or two independent SMARCC1 siRNAs against human or mouse SMARCC1 for 48 hours. Cell images were acquired and the cell lysates were Western blotted with anti-SMARCC1 and actin antibodies. Significance was indicated as two-tailed, unpaired, *t* test for (**a** and **b**). Values are expressed as mean ± SEM. *p<0.05, ***p<0.001. Scale bar: 200 μm.

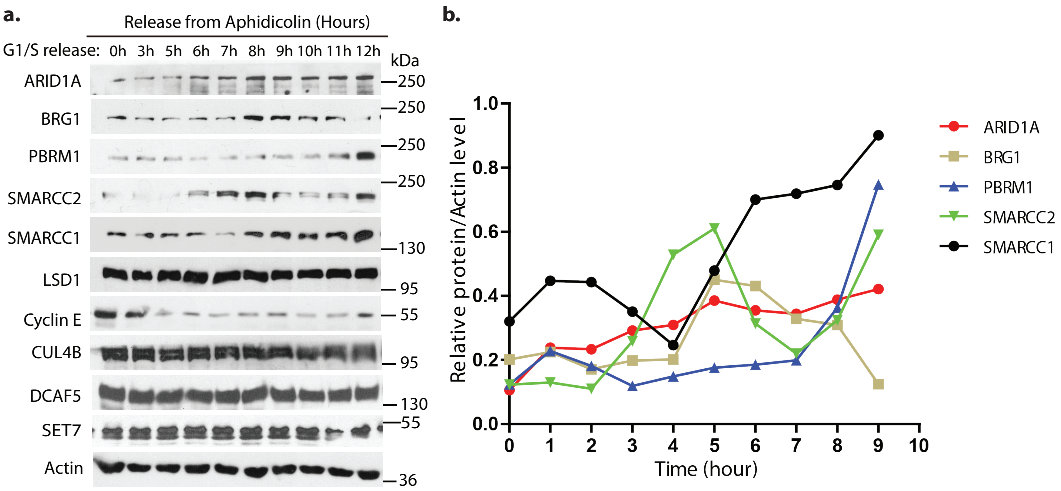

Supplementary Figure 9. Regulation of the mSWI/SNF proteins in the cell cycle. **a** and **b**. HeLa cells were synchronized by treating with 2.5 mM thymidine for 18 hours, releasing into fresh cell culture medium for 9 hours, and then treating again with 5 µg/ml aphidicolin for another 15 hours to synchronously arrest the cells at the G1/S border. Cells were washed and released into fresh culture medium without aphidicolin, and collected at various indicated time points. The levels of SMARCC1, ARID1A, BRG1, PBRM1, SMARCC2, cyclin E, LSD1, CUL4B, DCAF5, SET7, and actin were analyzed by Western blotting (**a**) and protein bands of ARID1A, BRG1, PBRM1, SMARCC2 and SMARCC1 in the cell cycle were quantified and plotted against the cell cycle progression time points (**b**).

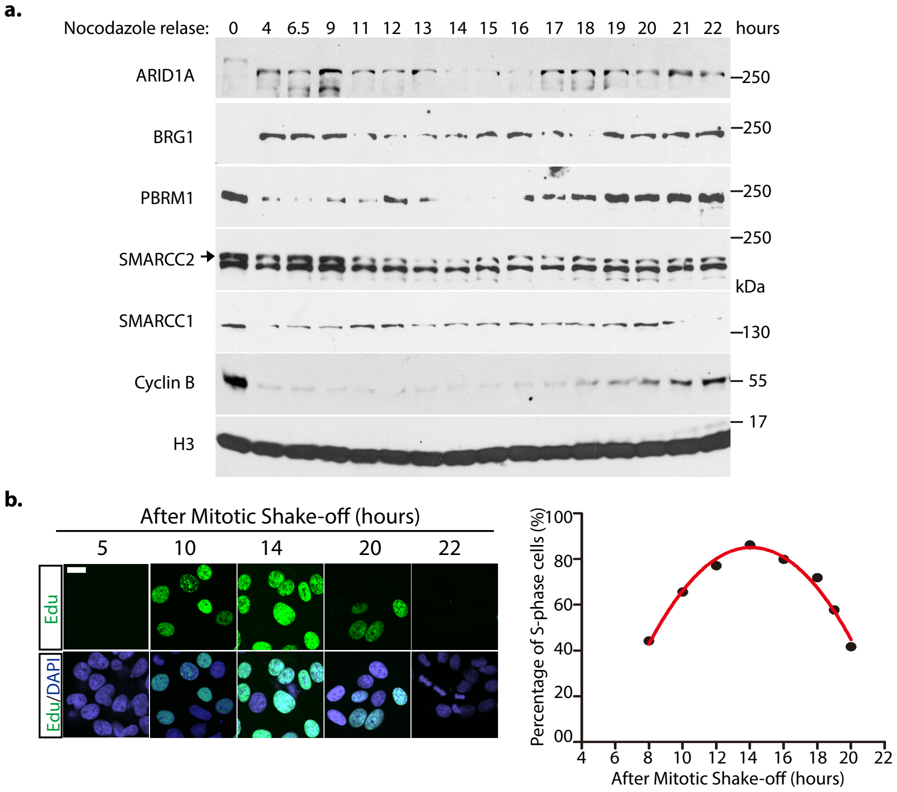

Supplementary Figure 10. Regulation of mSWI/SNF proteins in the cell cycle. **a**. HeLa cells were synchronized in mitosis by treating with 40 ng/ml nocodazole for 14 hours. The rounded-up mitotic cells were collected, washed, and released into fresh culture medium without nocodazole, and collected at various indicated time. The protein levels of ARID1A, BRG1, PBRM1, SMARCC2, SMARCC1, cyclin B, and histone H3 were analyzed by Western blotting. **b.** A fraction of cells in **a** was labeled with 5-ethynyl-2’-deoxyuridine (Edu), a nucleoside analogue of thymidine, to monitor S phase DNA replication, counter-stained with 4′,6-diamidino-2-phenylindole (DAPI) for nuclear DNA (left panel), and the percentages of cells with Edu incorporation were plotted (right panel). The Edu labeling indicates that S phase peaked at 14-15 hours post-mitotic release, whereas G2 and mitosis occurred at around 22 hours, indicated by the presence of mitotic meta-phase cells with DAPI staining. Scale bar: 20 μm.
