# Supplementary material for: The assembly of mammalian SWI/SNF chromatin remodeling complexes is regulated by lysine-methylation dependent proteolysis
Source: Nat Commun. 2022 Nov 5;13:6696. doi: 10.1038/s41467-022-34348-9 (PMC9637158; doi:10.1038/s41467-022-34348-9)
Supplement: Supplementary file 2 — Reporting Summary [file 41467_2022_34348_MOESM2_ESM.pdf]

nature portfolio

Corresponding author(s): Hui Zhang

Last updated by author(s): 2022/09/18

Reporting Summary

Nature Portfolio wishes to improve the reproducibility of the work that we publish. This form provides structure for consistency and transparency in reporting. For further information on Nature Portfolio policies, see our Editorial Policies and the Editorial Policy Checklist.

Statistics

For all statistical analyses, confirm that the following items are present in the figure legend, table legend, main text, or Methods section.

n/a	Confirmed	
☐	☒	The exact sample size (*n*) for each experimental group/condition, given as a discrete number and unit of measurement
☐	☒	A statement on whether measurements were taken from distinct samples or whether the same sample was measured repeatedly
☐	☒	The statistical test(s) used AND whether they are one- or two-sided<br>*Only common tests should be described solely by name; describe more complex techniques in the Methods section.*
☐	☒	A description of all covariates tested
☐	☒	A description of any assumptions or corrections, such as tests of normality and adjustment for multiple comparisons
☐	☒	A full description of the statistical parameters including central tendency (e.g. means) or other basic estimates (e.g. regression coefficient) AND variation (e.g. standard deviation) or associated estimates of uncertainty (e.g. confidence intervals)
☒	☐	For null hypothesis testing, the test statistic (e.g. *F*, *t*, *r*) with confidence intervals, effect sizes, degrees of freedom and *P* value noted<br>*Give P values as exact values whenever suitable.*
☒	☐	For Bayesian analysis, information on the choice of priors and Markov chain Monte Carlo settings
☒	☐	For hierarchical and complex designs, identification of the appropriate level for tests and full reporting of outcomes
☒	☐	Estimates of effect sizes (e.g. Cohen's *d*, Pearson's *r*), indicating how they were calculated

Our web collection on statistics for biologists contains articles on many of the points above.

Software and code

Policy information about availability of computer code

Data collection	No softwares and codes are used
Data analysis	No softwares and codes are used

For manuscripts utilizing custom algorithms or software that are central to the research but not yet described in published literature, software must be made available to editors and reviewers. We strongly encourage code deposition in a community repository (e.g. GitHub). See the Nature Portfolio guidelines for submitting code & software for further information.

Data

Policy information about availability of data

All manuscripts must include a data availability statement. This statement should provide the following information, where applicable:

- Accession codes, unique identifiers, or web links for publicly available datasets
- A description of any restrictions on data availability
- For clinical datasets or third party data, please ensure that the statement adheres to our policy

The authors declare that the data supporting the findings of this study are available within the paper and its supplementary information files. The data that support the findings of this study are also available from the corresponding author upon reasonable request.

Human research participants

Policy information about studies involving human research participants and Sex and Gender in Research.

Reporting on sex and gender	Not applicable
Population characteristics	Not applicable
Recruitment	Not applicable
Ethics oversight	Not applicable

Note that full information on the approval of the study protocol must also be provided in the manuscript.

Field-specific reporting

Please select the one below that is the best fit for your research. If you are not sure, read the appropriate sections before making your selection.

- [x] Life sciences
- [ ] Behavioural & social sciences
- [ ] Ecological, evolutionary & environmental sciences

For a reference copy of the document with all sections, see nature.com/documents/nr-reporting-summary-flat.pdf

Life sciences study design

All studies must disclose on these points even when the disclosure is negative.

Sample size	For animal genetic analyses, all the requested number of mice based on the calculation of the Sanger Mouse Genetics Project which requires 7 male and 7 female homozygous mice, generated by a heterozygous cross, and the suggestion that a best case scenario would require 14 mating pairs being assembled at the same point in time (PLOS ONE, December 2012 | Volume 7 | Issue 12 | e52410). We have used traditional statistical tests, such as a Student's t-Test or ANOVA, that control the false positive rate if factors such as body weight and batch do not affect the phenotype. Moreover, we have used Student's t-Test in our analyses as the Student's t-Test is the most powerful statistical test for a phenotypic difference in the means of two groups (wildtype and homozygous mutations). For all biochemical and cell biological analyses, almost all siRNA experiments or related investigation are repeated at least 3 times for similar results.
Data exclusions	The L3MBTL3 null embryos between E17.5-19.5 usually died and became disintegrated so the dead embryos were excluded from protein analyses.
Replication	Experiments were usually performed with at least three independent repeats (biological replicates) to ensure the results. To quantify protein loading in each Western blot analysis of a set of protein samples, the same protein samples were analyzed with three repeated loading experiments (technical replicates). For cell-based assays, triplicated repeats in the same set of cells (technical replicates) were measured and the experiments usually repeated in three independent experiments with independently cultured cells (biological replicates).
Randomization	In the experimental analyses of L3mbtl3 deletion mouse embryos or Lsd1 deletion mice, the investigators randomly analyzed wildtype, heterozygous and homozygous knockdown embryos.
Blinding	In the experimental analyses for examination of proteins in mouse embryos or mice, the investigators were unaware of the genotypes of the experimental embryos or animals.

Reporting for specific materials, systems and methods

We require information from authors about some types of materials, experimental systems and methods used in many studies. Here, indicate whether each material, system or method listed is relevant to your study. If you are not sure if a list item applies to your research, read the appropriate section before selecting a response.

Materials & experimental systems

n/a	Involved in the study
☐	☒ Antibodies
☐	☒ Eukaryotic cell lines
☒	☐ Palaeontology and archaeology
☐	☒ Animals and other organisms
☒	☐ Clinical data
☒	☐ Dual use research of concern

Methods

n/a	Involved in the study
☒	☐ ChIP-seq
☒	☐ Flow cytometry
☒	☐ MRI-based neuroimaging

Antibodies

Antibodies used	Anti-LSD1 (A300-215A), L3MBTL3 (A302-852), SMARCC2 (A301-038A), PBRM1 (A700-019), ARID1A (A301-040A), SMARCB1 (A301-087A), BRG1 (A301-087A), BRM (A301-015A), and SET7 (A301-747A) antibodies were purchased from Bethyl Laboratories; anti-BRG1 (49360) and anti-SMARCC1 (11956) were from Cell Signaling technology; anti-SMARCC1 (sc-32763) and actin (Sc-1616) antibodies were from Santa Cruz Biotechnologies. Anti-Flag, HA, and GFP antibodies were purchased from Sigma. Anti-DCAF5, anti-monomethylated K615, and monomethylated K482 antibodies were raised in rabbits and affinity purified from authors' laboratory.
Validation	All antibodies were verified when they were arrived or became available first time by Western blotting and/or immunoprecipitation followed by Western blotting on target proteins.

Eukaryotic cell lines

Policy information about cell lines and Sex and Gender in Research

Cell line source(s)	Human lung carcinoma H1299, cervical carcinoma HeLa, embryonic kidney carcinoma 293T, and mouse teratoma F9 cells were purchased from the American Type Culture Collection (ATCC) and cultured in RPMI-1640 or DMEM medium with 10% FBS and 1% antibiotics as described25,26,41. Mouse embryonic fibroblasts (MEFs) were generated from wildtype and L3mbtl3 deletion mutant embryos or CAGGCre-ERTM/LSD1fl/fl embryos (E12.5-E13.5). Mouse embryonic stem cells (CMTI-2, strain C57/ BL6J, passage 16) were obtained from Millipore-Sigma.
Authentication	Human 293T, HeLa, and H1299 cells were authenticated by the expression of protein markers such as p53, which is absent from H1299, p21, and p16Ink4A. Mouse F9 cells and mouse embryonic stem cells were authenticated by pluripotent protein markers such as SOX2, Oct4, and Nanog. Mouse embryonic fibroblasts were authenticated by expression of LSD1, L3mBTL3, actin and absence of SOX2.
Mycoplasma contamination	All cells were negative for the mycoplasma tests.
Commonly misidentified lines (See ICLAC register)	No

Animals and other research organisms

Policy information about studies involving animals; ARRIVE guidelines recommended for reporting animal research, and Sex and Gender in Research

Laboratory animals	The L3MBTL3 heterozygous mice (MBT-1-/+, B6;129-L3mbtl3tm1Tmiy) were originally provided by Dr. Miyazaki, Tokyo University. The LSD1fl/+ conditional mutant (B6.129-Kdm1a tm1.1Sho/J, stock No: 023969), transgenic actin-Cre-ER (CAGGCre-ERTM, B6.Cg-Tg (CAG cre/Esr1)5Amc/J, stock No: 004682), and transgenic Nestin-Cre (B6.Cg-Tg(Nes-cre)1Kln/J, stock No: 003771) mouse strains were obtained from Jackson Laboratory.
Wild animals	No wild animals were used in the experiments.
Reporting on sex	Equal number of male and female mice were identified by the presence of sex organs.
Field-collected samples	No field-collected or samples animals were used in the experiments.
Ethics oversight	All animal experiments including breeding, housing, genotyping, and sample collection were conducted in accordance with the animal protocols approved by the institutional Animal Use and Care Committee (IACUC) and complied with all relevant ethical regulations at University of Nevada, Las Vegas. All procedures were conducted according to the National Institutes of Health (NIH) Guide for Care and Use of Laboratory Animals. The UNLV IACUC is an AAALAC approved facility and meets the NIH Guide for the Care and Use of Animals.

Note that full information on the approval of the study protocol must also be provided in the manuscript.
